# Sonic Hedgehog and Notch Signaling Can Cooperate to Regulate Neurogenic Divisions of Neocortical Progenitors

**DOI:** 10.1371/journal.pone.0014680

**Published:** 2011-02-17

**Authors:** Richa K. Dave, Tammy Ellis, Melissa C. Toumpas, Jonathan P. Robson, Elaine Julian, Christelle Adolphe, Perry F. Bartlett, Helen M. Cooper, Brent A. Reynolds, Brandon J. Wainwright

**Affiliations:** 1 The Institute for Molecular Bioscience, The University of Queensland, Brisbane, Queensland, Australia; 2 Queensland Brain Institute, The University of Queensland, Brisbane, Queensland, Australia; 3 School of Biomedical Sciences, The University of Queensland, Brisbane, Queensland, Australia; The University of Hong Kong, China

## Abstract

**Background:**

Hedgehog (Hh) signaling is crucial for the generation and maintenance of both embryonic and adult stem cells, thereby regulating development and tissue homeostasis. In the developing neocortex, Sonic Hedgehog (Shh) regulates neural progenitor cell proliferation. During neurogenesis, radial glial cells of the ventricular zone (VZ) are the predominant neocortical progenitors that generate neurons through both symmetric and asymmetric divisions. Despite its importance, relatively little is known of the molecular pathways that control the switch from symmetric proliferative to differentiative/neurogenic divisions in neural progenitors.

**Principal Findings:**

Here, we report that conditional inactivation of Patched1, a negative regulator of the Shh pathway, in Nestin positive neural progenitors of the neocortex leads to lamination defects due to improper corticogenesis and an increase in the number of symmetric proliferative divisions of the radial glial cells. Hedgehog-activated VZ progenitor cells demonstrated a concomitant upregulation of *Hes1* and *Blbp*, downstream targets of Notch signaling. The Notch signaling pathway plays a pivotal role in the maintenance of stem/progenitor cells and the regulation of glial versus neuronal identity. To study the effect of Notch signaling on Hh-activated neural progenitors, we inactivated both *Patched1* and *Rbpj*, a transcriptional mediator of Notch signaling, in Nestin positive cells of the neocortex.

**Conclusions:**

Our data indicate that by mid neurogenesis (embryonic day 14.5), attenuation of Notch signaling reverses the effect of *Patched1* deletion on neurogenesis by restoring the balance between symmetric proliferative and neurogenic divisions. Hence, our results demonstrate that correct corticogenesis is an outcome of the interplay between the Hh and Notch signaling pathways.

## Introduction

The development of the mammalian neocortex requires the orchestration of numerous processes in a spatiotemporal manner resulting in the precise six-layered structure. Neurons and radial glia are the major cell types that constitute the vertebrate nervous system and are derived from the neuroepithelial cells, the primary neural progenitors [1]. After embryonic day (E)10, neuroepithelial cells give rise to radial glial cells, which then constitute the predominant progenitor population in the neocortex [2]. These radial glial cells serve both as neural precursors and migratory scaffolds. At the peak of neurogenesis, radial glial cells can undergo either symmetric proliferative divisions to maintain their own pool or can differentiate into either a basal progenitor (BP) that populates the newly formed subventricular zone (SVZ), or a neuron [3], [4], [5], [6]. These post-mitotic neurons subsequently migrate up the radial glial cells to form the cortical plate (CP). Corticogenesis thus requires the co-ordination of signaling pathways that provide mitogenic signals, positional information, migratory cues, differentiation signals, and a requirement for the precise regulation of symmetric/asymmetric division. Although some of these critical signaling pathways have been defined, in many examples we still lack a detailed understanding of the precise consequences of the interaction between them and the factors that influence the number and type of divisions a neocortical progenitor cell undergoes.

The Hedgehog signaling pathway plays a key role in the ventral patterning of the entire neuraxis, powerfully illustrated by the consequences of Sonic Hedgehog (Shh) secretion from the notochord during spinal cord development [7], [8]. Shh is also expressed in dorsal structures throughout the developing CNS, including the cerebellum, tectum and forebrain [9], [10], [11], [12]. Within the neocortex, Komada and colleagues have identified domains of Shh expression in the proliferative zones and post-mitotic neurons of the neocortex from E13.5–E15.5. Loss of function studies utilizing conditional Shh and Smoothened (Smo) alleles indicate that Hh signaling acts by regulating the cell cycle of VZ stem and progenitor cells and in the absence of an active pathway there is significantly reduced neurogenesis, patterning defects and a smaller dorsal telencephalon at E18.5 [12]. Consistent with these findings, gain of function studies *in vitro* have indicated that Hh signaling is mitogenic for both tectal and neocortical progenitors [11], [12].

Notch signaling plays a central role in the maintenance of neural stem cell phenotype [13], [14], [15]. During embryogenesis, Notch signaling promotes neural stem cell proliferation in the forebrain [14] by inhibiting neuronal fate and ablation of Notch signaling leads to premature differentiation of glial cells to neurons [16]. In addition, Notch signaling is essential for the maintenance of stem cell characteristics, possibly through the canonical Rbpj-mediated Notch signaling cascade [17]. Anthony *et al* have demonstrated that Blbp is a direct target of Notch signaling in radial glial cells thereby providing a direct link between active Notch signaling and the maintenance of the radial glial cell scaffold [18]. Despite its critical role in the maintenance of radial glial cells and the development of the mammalian neocortex, little is known to date about the interaction of Notch signaling pathway with other key developmental pathways in the spatiotemporal generation of the neocortex.

On the basis of published data, both the Notch and Hedgehog signaling pathways act concomitantly on dorsal forebrain development. In particular, both *in vitro* and *in vivo* studies addressing either Notch or Hh signaling suggest that the activity of both pathways is vital for the correct regulation of the stem cell niche, and the subsequent formation of the cortical layers [11], [12], [14], [17], [18], [19], [20], [21]. What is not known is whether these two pathways are capable of interacting to control neurogenesis. To address this issue we directly examined the interaction between Notch and Hedgehog signaling in the neocortex. Since the function of Hedgehog signaling in the neocortex appears to be the regulation of the size of stem/progenitor pool and loss of function studies lead to substantive patterning defects. As a general approach to defining Hh/Notch interaction, we used a gain of function mutation as our start point. We activated the Hedgehog pathway in neuroepithelial cells through the use of a conditional Ptc1 (*Ptc1^Lox/Lox^*) allele [22] in combination with a Nestin-promoter-driven Cre-recombinase mouse model (*Nestin^Cre^*) [23]. Ptc1 is the Hedgehog receptor and a negative regulator of Hedgehog signaling whilst Nestin is expressed in neuroepithelial cells from E10.5 into adult life but down regulated in differentiated cells derived from the neuroepithelium. Loss of Ptc1 from neuroepithelial cells creates a cell autonomous activation of the Hedgehog pathway. The *Ptc1^Lox/Lox^*;*Nestin^Cre^* embryos die at E15.5, therefore, the studies presented here were carried out on E14.5 or earlier developmental stages. To perturb Notch signaling we utilized a conditional allele of Rbpj (*Rbpj^Lox/Lox^*) [24], a common effector required for the activity of all four mammalian Notch receptors. When inactivated under the control of *Nestin^Cre^*, loss of Rbpj function results in premature differentiation of the stem cells of the dorsal forebrain [25]. Here we demonstrate that autonomous Hh signal activation in VZ progenitors leads to an increase in the symmetrical divisions of radial glial cells. The *Ptc1^Lox/Lox^;Nestin^Cre^* mice have an expansion of the VZ progenitors and improper patterning. Furthermore, Notch signaling targets *Hes1* and *Blbp* which were upregulated in the *Ptc1^Lox/Lox^;Nestin^Cre^* VZ suggesting that Hedgehog and Notch signaling may directly interact in the determination of radial glial cell fate in these animals. We tested this suggestion and determined that loss of Rbpj restored the number of neurogenic divisions to wild type levels in the *Ptc1^Lox/Lox^;Rbpj^Lox/Lox^;Nestin^Cre^* neocortex. We used *ex vivo* neurosphere based assays to define the proliferative compartment and demonstrate that *in vivo* activation of Hh signaling dramatically expands the number of stem and progenitor cells in the neocortex but loss of Rbpj attenuates this expansion to wild type levels. Overall, our results demonstrate that Hh signaling can co-operate with Notch signaling in the maintenance and expansion of neocortical stem cells.

## Materials and Methods

Mice with CNS-specific deletions of Ptc1 were obtained by breeding animals carrying the conditional *Ptc1* (*Ptc1^Lox/Lox^*) [22] or *Rbpj* allele (*Rbpj^Lox/Lox^*) [24] with Nestin-Cre (*Nestin^Cre^*) transgenic mice [23] and genotyping confirmation was carried out as outlined. All experimentation involving animals was approved by University of Queensland animal ethics committee and conformed to relevant ethical guidelines. Pregnant females were injected intraperitoneally with 0.1 ml/g (volume/body weight) of BrdU labelling reagent (Zymed, San Francisco, CA) for 2, 24, 48 or 72 hours prior to sacrificing.

### Histology, immunohistochemistry, *in situ* hybridisation and cell cycle analysis

Embryos were removed, fixed in 4% buffered paraformaldehyde overnight at 4°C, paraffin embedded and cut in 6 µm thick sections. Sections were stained with haematoxylin and eosin or stained for Nissl substance using standard histological techniques. Non-radioactive *in situ* hybridisation was performed using digoxigenin (DIG)-labelled cRNA probes as described [26]. The *Ptc1* probe was designed towards the C-terminal end of the stop codon, so that it detects *Ptc1* transcript. For immunohistochemistry, antigen retrieval of deparaffinised wax tissue sections was performed by boiling in antigen unmasking solution (Vector Laboratories, Burlingame, CA, USA) for 5 minutes. Sections were blocked in 5–10% horse serum, 2% BSA and 0.2% Triton-X in PBS prior to primary antibody incubation over night at 4°C as previously described [26]. Antibodies used were mouse anti-Nestin (1∶200, Chemicon, Temecula, CA), rabbit polyclonal anti-Tbr2 (1∶1000, Chemicon) and mouse anti-MAP2 (1∶500, Sigma Aldrich, St. Louis, MO). Slides were incubated with secondary antibodies for 1 hour at room temperature. Immunofluorescence was performed on coronal sections incubated with goat anti-Sox2 (1∶200, R&D Systems, Minneapolis, MN, USA); mouse anti-Nestin (1∶200, Chemicon, Temecula, CA) and rabbit anti-βIII Tubulin or TuJ1 (1∶300, Covance, Princeton, NJ). For immunofluorescence, a DAPI (4, 6-diamidino-2-phenylindole, Sigma Aldrich, St Louis, MO, USA, 1∶10000) counterstain was performed for 5 minutes prior to mounting with Fluorescence Mounting Media (Dako, Carpentaria, CA, USA). Fluorescent secondary antibodies used were anti-rabbit Alexa Fluor 488 (1∶250, Invitrogen), anti-rabbit Alexa Fluor 555 (1∶250, Invitrogen), anti-mouse Alexa Fluor 488 (1∶250, Invitrogen) and anti-goat Cy3 (1∶250, Abacus ALS Pty Ltd, Brisbane, Australia). Fluorescence images were obtained by using a laser confocal microscope (LSM Meta, Carl Zeiss). All images were taken in the medial neocortical region.

BrdU-immunohistochemistry was performed on paraffin-embedded sections as described [27]. Cell cycle length and exit studies were carried out as described [28] with minor modifications. Deparaffinised, rehydrated paraffin-embedded sections were boiled 3 times for 15 minutes each in antigen-retrieval buffer (Vector Laboratories, Burlingame, CA). Sections were blocked in 4% horse serum, 0.2% Triton X-100, 1% BSA in PBS for 60 minutes followed by primary antibody incubation overnight at 4°C with mouse anti-BrdU (1∶150, BD Biosciences, San Jose, CA) or rabbit anti-Ki67 (1∶200, Novocastra Laboratories, Newcastle upon Tyne, UK) followed by incubation for 1 hour at room temperature with fluorescent labelled secondary antibodies: goat anti-mouse Alexa Fluor 488 (1∶200) or goat anti-rabbit Cy3 (1∶250). Percentages of two fields of randomly selected 50 Ki67^+^/BrdU^+^ or 50 BrdU^+^/Ki67^−^ cells from each neocortex (n = 3) was compared by unpaired Student's t-test.

### Neurosphere cultures

Neocortices were dissected from E14.5 *Ptc1* or *Rbpj* mutant mice, filtered through a 40 µm nylon cell strainer (Stem Cell Technologies Inc. Vancouver, BC) and plated directly. For the SFUA (Sphere forming unit assay), cells were plated at a density of 2×10^4^ cells/well in 96 well plates containing Neuro Cult complete medium (Stem Cell Technologies) with EGF (20 ng/ml) [29]. For each individual sample, the assay was set up in triplicates. The numbers of spheres per well were counted 7 days post plating and were plotted as the percentage of the number of spheres obtained per 20,000 cells plated per well. Statistical analysis was performed using GraphPad Prism 4 for unpaired Student's t-test with Welch's correction. For passaging, neurospheres were harvested and dissociated in 0.05% trypsin (Gibco) followed by addition of trypsin inhibitor. Resuspended cell pellets were then replated at a density of 2×10^5^ cells/ml. Cell counts at every passage were used for growth curve analysis. For analysis of neurosphere replating assays in GraphPad Prism 4, expansion numbers obtained per passage were transformed into LOG and linear regression analysis was performed on resulting curves. Student's t-test analysis was used to determine significant differences between slopes.

### Pair cell assay

The Pair Cell Assay was performed as described [30], [31]. Briefly, the neocortices from E14.5 embryos were dissected into HBSS (Gibco, Invitrogen, Carlsbad, CA) and enzymatically dissociated using 0.125% trypsin (Gibco) at 37°C for 10 minutes and titrated with a fire polished glass Pasteur pipette to generate single cell suspension. The cell suspension was passed through a 40 µm cell strainer (BD Biosciences), rinsed 3 times in HBSS followed by gentle centrifugation. The cell pellet was resuspended in serum-free pair cell medium [32]. Single cells were plated on poly-d-lysine (Sigma Aldrich) coated coverslips at a cell density of about 50,000 cells per well and cultures were maintained for 24 hours. For immunofluorescence studies, the cells were fixed in 4% PFA at 37°C for 30 minutes, before blocking for 30 minutes at room temperature in 0.5% BSA in PBS. The fixed cells were then incubated with rabbit monoclonal anti-TuJ1 (1∶1000; Covance) and guinea pig polyclonal anti-GLAST (1∶200; Chemicon) primary antibodies at room temperature for 1 hour, followed by incubation with secondary antibodies for 1 hour: chicken anti-rabbit Alexa Fluor 488 and goat anti-guinea pig Alexa Fluor 594 (1∶250, Molecular Probes) and DNA was counterstained with DAPI (Sigma, St. Louis, MO). Coverslips were mounted on slides and imaged using laser confocal microscope (LSM Meta, Carl Zeiss). The number of progenitor pairs undergoing proliferative or neurogenic/differentiative cell divisions was determined by counting 100 pair of the cells per mutant and wild type samples (n = atleast 3) and the data was analysed using GraphPad Prism 4 software.

### VZ microdissection and Real-Time PCR

E14.5 embryos were dissected and embedded immediately in OCT medium. 50 µm sagittal sections were cut and mounted onto PALM Membrane Slides (Carl Zeiss Pty Ltd). Slides were then fixed and stained with Cresyl Violet according to the LCM Staining Kit (Ambion, Austin, TX). The neocortical ventricular zones were microdissected under a dissecting microscope using a sterile scalpel blade and pooled. Total RNA was extracted using an RNeasy Mini Kit (Qiagen) followed by DNase treatment using RNase Free DNase set (Qiagen, Germantown, MD).

For real time RT-PCR analysis, first strand cDNA was synthesized from 200 ng total RNA using Superscript III Reverse Transcriptase (Invitrogen). Real-time PCR reactions were prepared in triplicate on ABI Prism SDS 7000 using a 20 µl reaction mixture with SYBR Green PCR Master Mix (Applied Biosystems, Foster City, CA). The raw data for *Ptc1* SYBR Green RT-PCR was normalised based on the expression of *Hprt*. Primers used in this analysis were: *Ptc1* (Forward: 5′ GGC AAG TTT TTG GTT GTG GGT C 3′ and Reverse: 5′ CCT CTT CTC CTA TCT TCT GAC GGG 3′). *mHprt* (Forward: 5′GCA GTA CAG CCC CAA AAT GG 3′ and Reverse: 5′ AAC AAA GTC TGG CCT GTA TCC AA 3′). Inventoried assay on demand was used for *Gli1* (Mm00494645_m1, Applied Biosystems), *Ptc1* (Mm00436029_m1, Applied Biosystems; for detection of total *Ptc1* transcript), *Hes5* (Mm00439311_g1, Applied Biosystems), *Blbp* (Mm00445225_m1, Applied Biosystems) and *Hes1* (Mm01342805_m1, Applied Biosystems) RT PCR reactions. Reactions were prepared in duplicates on ABI Prism SDS 7500 using a 20 µl reaction mixture with Taqman Master Mix (Applied Biosystems). The raw data was normalised based on the expression of mouse *Gapdh* (VIC-labelled assay on demand, Applied Biosystems).

### Microdissected VZ genotyping

To confirm for the recombined *Ptc1* exon 3 deleted transcript exon 2/exon 6 genotyping PCR was used. Cre-mediated recombination results in a functionally null Ptc1 protein truncated 13 amino acids after the first transmembrane domain [22]. Primers used for this analysis were: *Ptc1* exon 2 (Forward: 5′ CAC CGT AAA GGA GCG TTA CCT A 3′) and *Ptc1* exon 6 (Reverse: 5′ TGG TTG TGG GTC TCC TCA TAT T 3′). Genotyping of the wildtype *Ptc1* transcript would produce a 450 bp band, upon Cre-mediated recombination exon3 is excised and the resulting transcript is 250 bp.

## Results

### Activation of Hedgehog signaling in Nestin^+^ cells by Patched1 deletion leads to altered neocortical patterning

**Figure 1:**
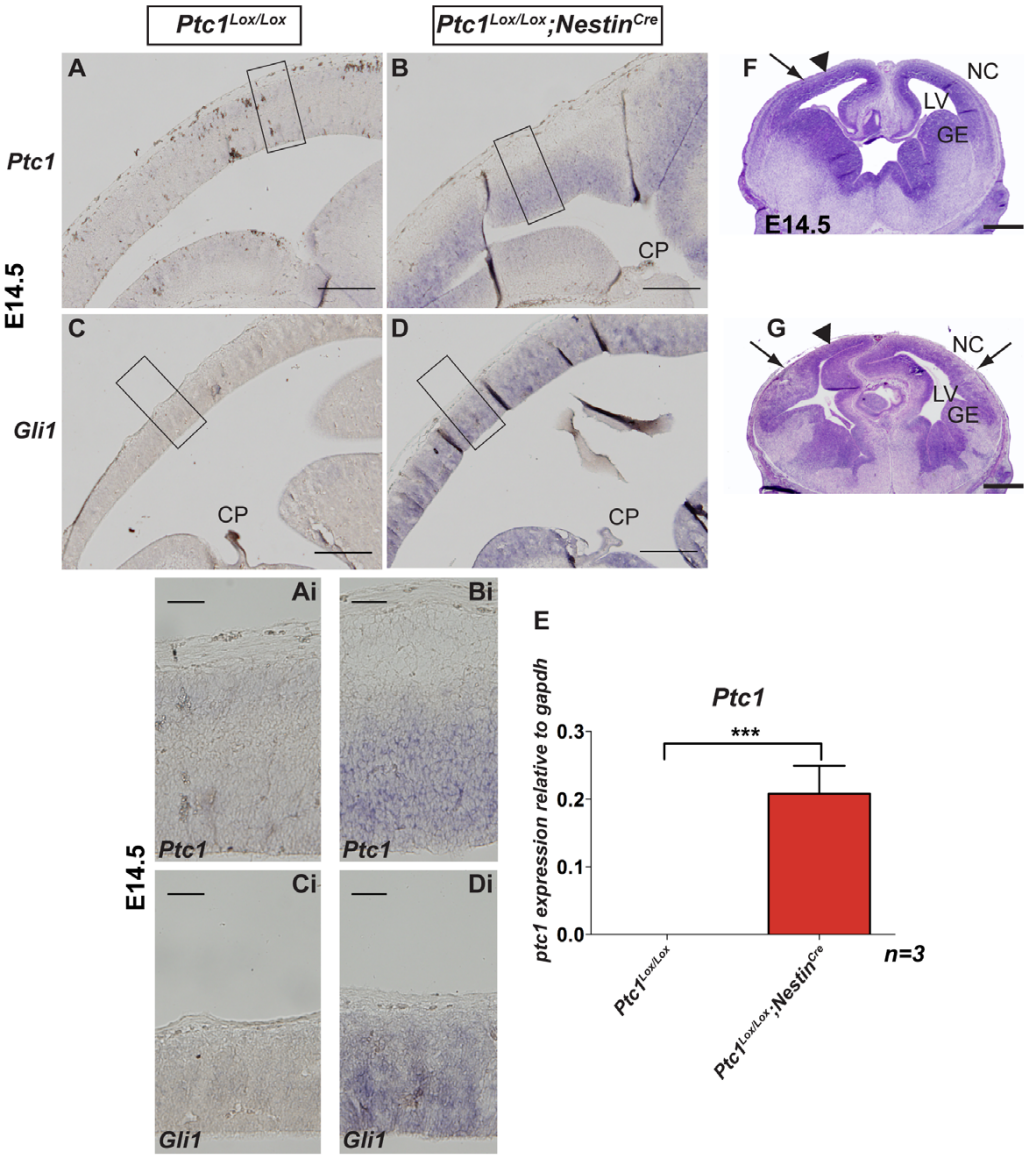
Activated Hh pathway leads to neocortical defects in *Ptc1^Lox/Lox^;Nestin^Cre^* dorsal brain. *In situ* hybridisation on E14.5 sagittal sections demonstrating that both *Ptc1* (**A, B, Ai, Bi)** and *Gli1* (**C,** **D, Ci,** **Di**) transcripts are upregulated in VZ of *Ptc1^Lox/Lox^;Nestin^Cre^* neocortex compared to wild type *Ptc1^Lox/Lox^* littermate. (**Ai–Di**): shows a higher magnification of the wild type and mutant neocortex in **A–D** (boxed area). **E**: Taqman RT-PCR for detection of total *Ptc1* transcript in microdissected VZ of wild type and mutant neocortex. The expression of *Ptc1* was upregulated in E14.5 *Ptc1^Lox/Lox^;Nestin^Cre^* VZ compared to *Ptc1^Lox/Lox^* VZ, due to negative feedback mechanism. The data was normalised based on the expression of *Gapdh* and is presented as three independent pooled samples of each genotype. (**F, G**): Haematoxylin and Eosin stained coronal sections of E14.5 neocortex. *Ptc1^Lox/Lox^;Nestin^Cre^* neocortex is extensively folded and GE are distorted compared to *Ptc1^Lox/Lox^* (**F**). The regions of thickened neuroepithelium in the mutant are not consistent even in the same rostral/caudal plane (arrows) with regions of normal thickness also present (arrowheads). Scale bars (**A–D, F–G**), 100 µm; (**Ai–Di**), 50 µm. Abbreviations: CP, choroid plexus; GE, ganglionic eminence; LV, lateral ventricle; NC, neocortex; VZ, ventricular zone.

To demonstrate activation of Hh signaling in mice homozygous for Nestin^+^ cell specific inactivation of the *Ptc1* gene *(Ptc1^Lox/Lox^;Nestin^Cre^* ), the expression of *Ptc1* and *Gli1* transcripts [33] was analysed. Both *Ptc1* and *Gli1* are universal Hh targets and upon *Ptc1* inactivation in *Ptc1^Lox/Lox^;Nestin^Cre^* mice an upregulation of both *Ptc1* and *Gli1* transcripts in the VZ of the developing E14.5 neocortex was observed (Fig. 1B, 1D, 1Ai–Di). Although we were unable to detect *Ptc1* and *Gli1* expression in the neocortex of *Ptc1^Lox/Lox^* mice by section *in situ* hybridisation (Fig. 1A, 1C), total *Ptc1* transcript was detected by Taqman RT-PCR in microdissected VZ (Fig. 1E) and the deleted or mutant transcript was detected by SYBR Green RT-PCR (Fig. S1B). These results demonstrate that Ptc1 is normally expressed in the cortical VZ neural progenitors at low levels that are sufficient to reduce the Hh pathway activity and a consequence of Ptc1 inactivation is the generation of a robust physiological cell-autonomous Hh response.

Defects were observed in dorsal forebrain and midbrain development in *Ptc1^Lox/Lox^;Nestin^Cre^* embryos beginning at E11.5 (data not shown). In E14.5 *Ptc1^Lox/Lox^;Nestin^Cre^* embryos, the neocortex, hippocampus, medial pallium and the ganglionic eminences were distorted. In contrast to the smooth surface of the *Ptc1^Lox/Lox^* neocortex (Fig. 1F), the neuroepithelial surface of the *Ptc1^Lox/Lox^;Nestin^Cre^* neocortex (Fig. 1G) was extensively irregular and folded. This results in regions of decreased cortical thickness and regions of folding. This irregularity was apparently not associated with differences in the rostral/caudal positioning (Fig. S2A–F).

### Sustained Hedgehog signaling in VZ progenitors promotes symmetric proliferative divisions of radial glial cells

**Figure 2:**
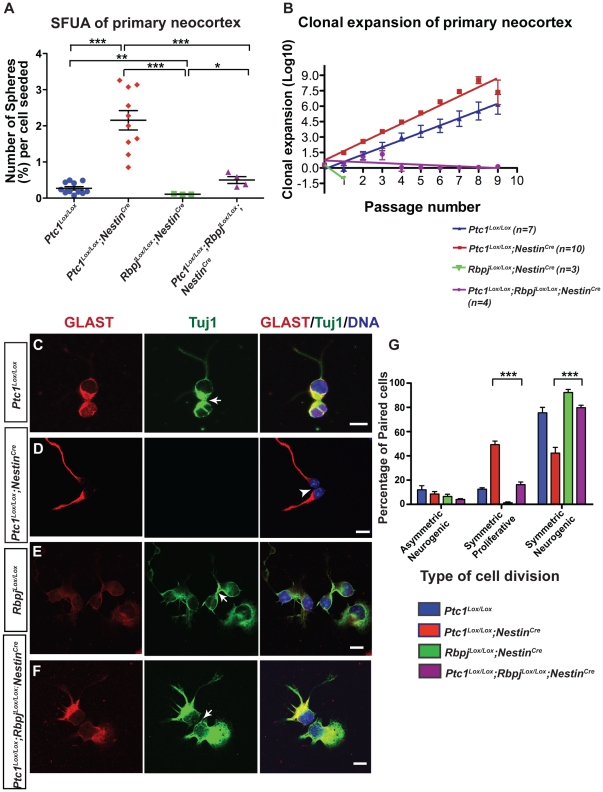
Hh pathway activation expands the population of cells with stem cell-like properties and promotes symmetric proliferative divisions of radial glial cells. (**A**) E14.5 *Ptc1^Lox/Lox^;Nestin^Cre^* neocortex (n = 10) showed an 8-fold increase (***p<0.0001) in the number of primary neurosphere colonies per cell seeded compared to littermate controls (n = 11). For *Rbpj^Lox/Lox^;Nestin^Cre^* and *Ptc1^Lox/Lox^;Rbpj^Lox/Lox^;Nestin^Cre^* samples, n = 3 and n = 4 respectively. * and ** p<0.005 by unpaired Student's t-test (with Welch's correction). (**B**) Clonal expansion of neurospheres derived from E14.5 neocortex. Cell numbers obtained at each passage were transformed into LOG and linear regression analysis was performed on resulting curves. The rate of clonal expansion of the neurospheres is represented as the slope of the line. The number of stem cells was significantly increased in *Ptc1^Lox/Lox^;Nestin^Cre^* neurospheres compared to *Ptc1^Lox/Lox^* neurospheres (slope: 0.8877±0.06774 vs 0.7060±0.03302, p<0.05). However, *Rbpj^Lox/Lox^;Nestin^Cre^* and *Ptc1^Lox/Lox^;Rbpj^Lox/Lox^;Nestin^Cre^* neurospheres showed a significant reduction in stem cells compared to the wild type control (slope: −1.337±3.400e+038, p<0.0001 and slope: −0.08099±0.04816, p value compared to control <0.0001, respectively). Error bars indicate standard deviation (**A, B**). (**C–F**): Co-immunofluorescence of GLAST (red) and TuJ1 (green) on neocortical progenitors isolated from E14.5 *Rbpj* and *Ptc1* mutant and wild types neocortices. Cells were counterstained with DAPI (blue). Arrows (in **C, E, F**) indicate TuJ1^+^ cells and arrowheads (**D**) indicate GLAST^+^ radial glial cells. RG cells from *Ptc1^Lox/Lox^;Nestin^Cre^* neocortex rarely differentiate into TuJ1^+^ neurons after 24 hours (**D, G**), whereas concomitant loss of the *Rbpj* and *Ptc1* alleles results in an increased rate of neurogenesis in *Ptc1^Lox/Lox^;Rbpj^Lox/Lox^;Nestin^Cre^* neocortical cells (**F, G**). On the other hand, cells from *Rbpj^Lox/Lox^;Nestin^Cre^* neocortex mostly differentiate into TuJ1^+^ neurons after 24 hours. Also, (**G**): Quantitative analysis for GLAST and TuJ1 revealed that the percentage of RG cells undergoing symmetric neurogenic divisions was significantly increased (*p*<0.05) in the *Ptc1^Lox/Lox^;Rbpj^Lox/Lox^;Nestin^Cre^* compared to the *Ptc1^Lox/Lox^;Nestin^Cre^* neocortices. On the other hand, there was a reduction in the number of symmetric proliferative cell divisions in *Ptc1^Lox/Lox^;Rbpj^Lox/Lox^;Nestin^Cre^* compared to *Ptc1^Lox/Lox^;Nestin^Cre^* (p<0.05) samples. Bars represent standard errors. ***p<0.0001. Scale bar (**C–F**), 10 µm.

*In vivo* and *in vitro* loss of function studies have shown that Hh signaling is required for the maintenance of stem cells in the developing neocortex [9], [11], [12], [21], [34]. To directly address the consequences of sustained Hh signaling in neocortical precursors, the neurosphere culture assay was utilised to test for stem and progenitor cell properties [29]. We observed a substantial (approximately 8-fold) increase in sphere forming potential of cells derived from the *Ptc1^Lox/Lox^;Nestin^Cre^* neocortex (2.154±0.267%) compared to *Ptc1^Lox/Lox^* neocortex (0.27±0.047%, p<0.0001,) (Fig. 2A) indicating that Hh pathway activation *in vivo* can regulate the total number of stem and progenitor cells. Next, the neurospheres were passaged multiple times to select against lineage-committed progenitor cells not capable of self-renewal. The rate of clonal expansion of the neurospheres is represented as the slope of the line. There was a clear difference in the rate of fold increase between *Ptc1^Lox/Lox^* and *Ptc1^Lox/Lox^;Nestin^Cre^* neocortical samples (clonal expansion of 0.7060±0.03302 vs. 0.8877±0.06774, p<0.05) (Fig. 2B) indicating that Hh pathway activation is mitogenic for cells that have a capacity to self-renew over an extended period of time. We also investigated whether the enlargement of the neocortical VZ progenitor population was a result of changes in cell cycle kinetics due to *Ptc1* inactivation. If enlargement of the progenitor pool is due to shortening of the cell cycle, the proportion of progenitor cells labelled by a single 2-hour pulse of BrdU in the total population of cycling progenitors (Ki67^+^) would increase. Indeed, a significant shortening of the cell cycle in the mutant E14.5 neocortex was detected confirming that *Ptc1* inactivated progenitor cells divide significantly faster than wild type progenitors (Fig. S3B). In the *Ptc1^Lox/Lox^;Nestin^Cre^* neocortex, the proportion of VZ progenitors co-labelled with BrdU/Ki67 was approximately two-fold greater when compared to wild type littermates (Fig. S3D). Altogether, our results suggest that Hh pathway activation can regulate neocortical stem and/or progenitor cell divisions and the rate of cell division *in vivo*.

The expansion of the stem/progenitor population in the *Ptc1* inactivated neocortex suggested a possible perturbation of number and types of differentiative divisions that radial glial cells undergo. In order to investigate this we utilized the clonal pair cell assay [30], [31] which was performed on cells derived from the E14.5 neocortex of *Ptc1^Lox/Lox^;Nestin^Cre^* and *Ptc1^Lox/Lox^* mice. Immunofluorescence studies performed using GLAST (radial glial cells) and TuJ1 (post-mitotic neurons) antibodies revealed a marked reduction in the number of neurogenic divisions in cells from the *Ptc1^Lox/Lox^;Nestin^Cre^* neocortex compared to the wild type littermates (Fig. 2C, 2D, 2G). GLAST-positive neocortical cells divide either to generate two GLAST-positive radial glial cells (symmetric proliferative), a GLAST-positive radial glial cell plus a TuJ1-positive neuron (asymmetric neurogenic) or two TuJ1-positive neurons (symmetric neurogenic). The majority of the *Ptc1^Lox/Lox^;Nestin^Cre^* paired cells divided to generate two GLAST-positive radial glial cells and rarely differentiated into TuJ1-positive neurons (Fig. 2D). Whereas, *Ptc1^Lox/Lox^* neocortical cells were routinely GLAST^+^/TuJ1^+^ suggesting that these were early neurons that had recently differentiated from a radial glial cell (Fig. 2C). Quantification of the mode of cell division of paired cells stained with GLAST and TuJ1 revealed an approximate 4-fold increase (p<0.05) in the number of symmetric proliferative cell divisions and a subsequent 1.8 fold decrease (p<0.05) in the number of cells undergoing symmetric neurogenic cell divisions in the mutant *Ptc1^Lox/Lox^;Nestin^Cre^* neocortices (Fig. 2G). These data were further supported by immunocytochemical analysis of *Ptc1^Lox/Lox^;Nestin^Cre^* neocortex using antibodies against Nestin, MAP2 and TuJ1 (Fig. S4A–D, Fig. S6A–D). Nestin is expressed in both neuroepithelial and radial glial cells but not basal progenitors [35], [36], and its expression was found to be expanded and more intense in the *Ptc1^Lox/Lox^;Nestin^Cre^* neocortex compared to the *Ptc1^Lox/Lox^* neocortex. The expansion of the Nestin domain is consistent with the observed increase in proliferation. A population of MAP2^+^ neurons were observed in the E12.5 *Ptc1^Lox/Lox^;Nestin^Cre^* neocortex, indicating that prior to *Ptc1* inactivation at E11.5/E12.5 (although the expression of Nestin is turned on at E10.5, it takes 24–48 hours to result in Patched1 inactivation), neuronal differentiation commenced normally (also see Fig. S4E–H). Radial glial cells can also differentiate into a Tbr2^+^ basal progenitor cell through asymmetric/symmetric differentiative divisions, reviewed in [37], which then undergoes one symmetric division to yield two neurons. The number of Tbr2^+^ cells were markedly reduced in the *Ptc1^Lox/Lox^;Nestin^Cre^* neocortex compared to wild type (Fig. S3E–G), further supporting the notion that the decrease in neuronal differentiation is a result of both a failure of radial glial cells to generate neurons directly and indirectly through basal progenitors. Overall these data indicate that the expansion of the proliferative compartment in the *Ptc1^Lox/Lox^;Nestin^Cre^* neocortex is likely the result of an increase in the number of symmetrical divisions of radial glial cells at the expense of neurogenic divisions.

### Ablation of Notch signaling in Hh-activated neural progenitors results in disruption of the neocortical structure

**Figure 3:**
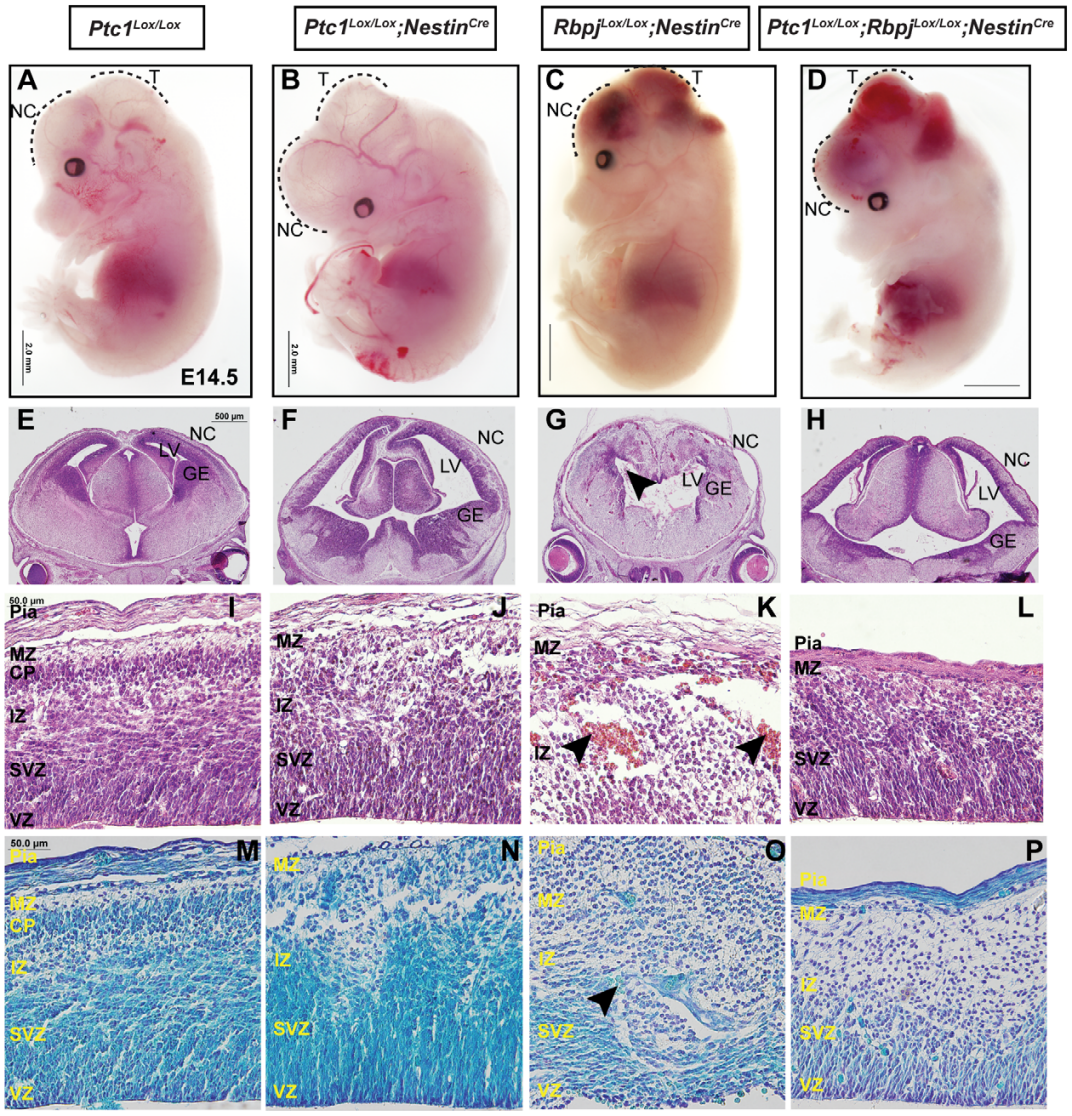
Hedgehog and Notch signaling are necessary for corticogenesis. Bright field microscopy images of whole mounts of E14.5 (**A–D**). Haematoxylin and Eosin stained coronal sections of E14.5 forebrains (**E–L**). (**I–L**): magnified views of (**E–H**). Nissl-stained coronal sections of E14.5 neocortices (**M–P**). In the *Ptc1^Lox/Lox^;Nestin^Cre^* neocortex, the VZ is expanded and the SVZ and CP cannot be identified (**J, N**) compared to the *Ptc1^Lox/Lox^* neocortex (**I, M**). The *Rbpj^Lox/Lox^;Nestin^Cre^* neocortex is highly disorganised, lacks diencephalon and contains blood clots (arrowheads) within the neocortex and the LV. (**G, K, O**). *Ptc1^Lox/Lox^;Rbpj^Lox/Lox^;Nestin^Cre^* neocortex (**H, L, P**) is thinner than the wild type neocortex, but the VZ and SVZ is comparable to the wild type. Abbreviations: CP, cortical plate; GE, ganglionic eminence; IZ, intermediate zone; LV, lateral ventricle; MZ, marginal zone; NC, neocortex; SVZ, sub-ventricular zone; T, tectum; VZ, ventricular zone. Scale bar (**A–D**), 2.0 mm; (**E–H**), 500 µm; (**I–P**), 50 µm.

**Figure 4:**
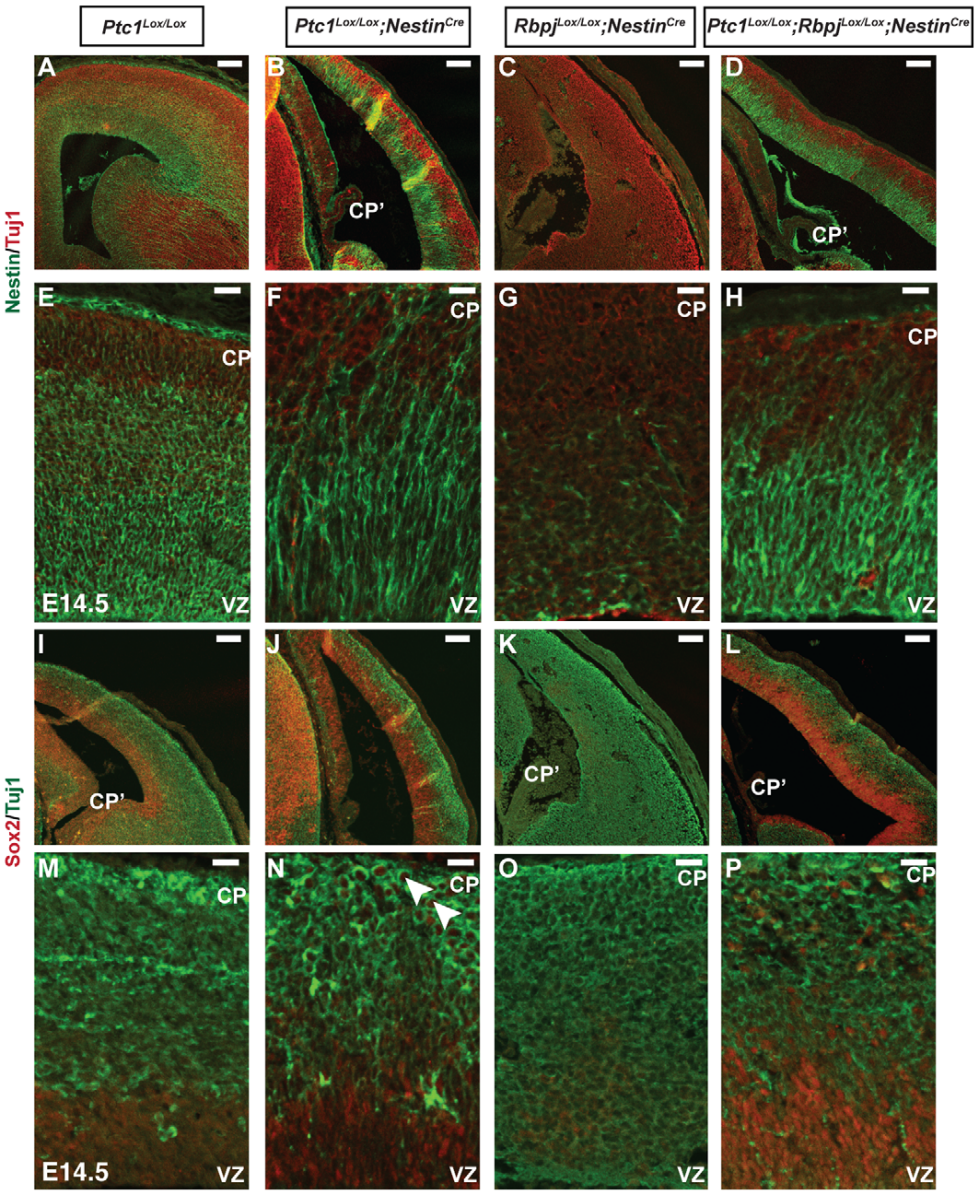
Loss of Rbpj leads to disruption of neocortical patterning. Immunostaining of E14.5 neocortex using TuJ1 (red) (**A–H**); (green), (**I–P**) and Nestin (green) (**A–H**) or Sox2 (red) (**I–P**) antibodies. High magnification confocal images of the medial region of coronal sections from **A–D, I–L** are shown in **E–H, M–P** respectively. The zone of cells that are Sox2 and Nestin positive is expanded in *Ptc1^Lox/Lox^;Nestin^Cre^* and *Ptc1^Lox/Lox^;Rbpj^Lox/Lox^;Nestin^Cre^* samples. Sox2^+^ cells were even present in the CP (arrowheads in **N**). TuJ1^+^ cells were present throughout the *Rbpj^Lox/Lox^;Nestin^Cre^* neocortex, from the VZ to the pial surface. Abbreviations: CP, cortical plate; CP', choroid plexus; VZ, ventricular zone. Scale bar (**A–D, I–L**), 100 µm; (**E–H, M–P**), 20 µm.

Several reports have suggested the importance of Notch signaling in controlling asymmetric/symmetric divisions in neural stem and progenitor cells (reviewed in [30], [38]). Both *in vivo* and *in vitro* studies have established that Notch1 and Notch3 activation promote radial glial cell fate [14], [39] and inactivation of Notch1 signaling leads to precocious neuronal differentiation [40]. Since our data indicate that Hedgehog pathway activation may play a similar role in the same cell types we next asked whether Notch and Hedgehog signaling interact in the regulation of homeostasis in the VZ. To examine whether the increase in the number of radial glial cells in *Ptc1^Lox/Lox^;Nestin^Cre^* neocortex required Notch signaling, inactivation of *Rbpj* and *Ptc1* was carried out concomitantly in Nestin^+^ neural progenitors. Forebrains from the control genotypes (*Ptc1^Lox/Lox^*, *Ptc1^Lox/Lox^;Rbpj^+/Lox^*, *Ptc1^Lox/Lox^;Rbpj^Lox/Lox^*) were examined and demonstrated normal neocortical development, although only *Ptc1^Lox/Lox^* neocortex samples are presented as a control in Figures 3 and 4. Whole mounts of E14.5 embryos from the *Rbpj^Lox/Lox^;Nestin^Cre^* and *Ptc1^Lox/Lox^;Rbpj^Lox/Lox^;Nestin^Cre^* mice showed extensive hemorrhage in the neocortex and tectum (Fig. 3C, 3D, Fig. S5), possibly due to loss of Rbpj from Nestin^+^ endothelial cells, however this phenotype has not been reported for the Rbpj-conditional knockout mice generated using inducible Nestin-Cre [41]. Haematoxylin and Eosin staining of E14.5 forebrains clearly demonstrated a patterning defect in the *Ptc1*-inactivated, *Rbpj*-inactivated and the double mutants (Fig. 3E–H). The diencephalon was missing in the *Rbpj^Lox/Lox^;Nestin^Cre^* forebrains (Fig. 3G) and the lateral ventricles of the *Ptc1^Lox/Lox^;Rbpj^Lox/Lox^;Nestin^Cre^* forebrains were enlarged (Fig. 3H). The neocortex of double mutant mice was much thinner and the neocortical structure was disrupted in contrast to the wild type neocortex, however, the VZ/SVZ was reduced compared to the *Ptc1*-deleted neocortex (Fig. 3L, 3P).

Precocious neuronal differentiation has been reported in mice with inactivated Notch signaling components [16], [42]. Moreover, it has been well documented that Notch signaling is instrumental in promoting stem/glial cell fate. To assess the effect of loss of Rbpj on neocortical neurogenesis at E14.5, we examined the expression of neuronal marker TuJ1 in conjunction with Nestin, which marks neuroepithelial and radial glial cells or Sox2. Interestingly, the zone of Nestin^+^ cells was expanded in *Ptc1^Lox/Lox^;Nestin^Cre^* and *Ptc1^Lox/Lox^;Rbpj^Lox/Lox^;Nestin^Cre^* neocortices compared to the wild type neocortex, with Nestin^+^ cells even present in the region of the cortical plate (Fig. 4F, 4H; Fig. S4B, S6B, S6D). On the other hand, *Rbpj^Lox/Lox^;Nestin^Cre^* neocortex had fewer Nestin^+^ cells (Fig. 4C, 4G). Also, as previously reported, TuJ1^+^ neurons were observed across the entire neocortex, from the ventricular surface to the pial surface in the *Rbpj^Lox/Lox^;Nestin^Cre^* forebrains (Fig. 4G). These results indicate that the delicate balance between proliferative and neurogenic divisions of neural precursors is disturbed as a result of over-activation of the Hh pathway and loss of Notch activity.

As Sox2 expression has been associated with actively dividing undifferentiated neural progenitor/stem cells and loss of Sox2 has been shown to be responsible for premature neuronal differentiation [43], [44], [45], we examined whether Sox2 expression was altered in Hh-activated and Rbpj-deleted neocortices. Sox2 expression was not restricted to the ventricular zone of *Ptc1^Lox/Lox^;Nestin^Cre^* and *Ptc1^Lox/Lox^;Rbpj^Lox/Lox^;Nestin^Cre^* neocortices with Sox2^+^ cells present in the more differentiated cells of the cortical plate in *Ptc1^Lox/Lox^;Nestin^Cre^* neocortex (arrowheads, Fig. 4N), suggesting a failure of progenitor cells to exit cell cycle and differentiate before migration. In support of our observation for Nestin staining, *Rbpj^Lox/Lox^;Nestin^Cre^* neocortex showed a dramatic reduction in Sox2^+^ cells in the ventricular zone (Fig. 4O, Fig. S6). Overall, due to disruption of the neocortical structure as a result of Hh pathway activation and deletion of *Rbpj* in the *Ptc1^Lox/Lox^;Nestin^Cre^*, *Rbpj^Lox/Lox^;Nestin^Cre^* and *Ptc1^Lox/Lox^;Rbpj^Lox/Lox^;Nestin^Cre^* neocortex, the layer-specific expression of Nestin, Sox2 and TuJ1 was perturbed. However, a distinct expansion of the Sox2 and Nestin domain was noted in the *Ptc1^Lox/Lox^;Nestin^Cre^* and *Ptc1^Lox/Lox^;Rbpj^Lox/Lox^;Nestin^Cre^* samples.

### Disruption of Notch signaling promotes symmetric neurogenic divisions and results in loss of putative neural stem cells in the *Ptc1^Lox/Lox^;Rbpj^Lox/Lox^;Nestin^Cre^* neocortex

To examine whether the imbalance in the number of neurons versus radial glia in the Ptc1-deleted and Rbpj-deleted neocortices was due to a shift in the number of symmetric proliferative divisions as a consequence of loss of Rbpj, the clonal pair cell assay was performed on cells from E14.5 *Ptc1^Lox/Lox^;Rbpj^Lox/Lox^;Nestin^Cre^* and *Rbpj^Lox/Lox^;Nestin^Cre^* mice. Immunostaining performed using GLAST and TuJ1 indicated that the majority of GLAST^+^ cells from *Rbpj^Lox/Lox^;Nestin^Cre^* neocortex undergo symmetric neurogenic divisions to expand the pool of post-mitotic neurons (Fig. 2E, 2G), observed previously across the entire neocortex (Fig. 4C, 4G) in accordance with the published data [16]. The number of radial glial cells undergoing symmetric neurogenic divisions was approximately double in *Ptc1^Lox/Lox^;Rbpj^Lox/Lox^;Nestin^Cre^* neocortex compared to *Ptc1^Lox/Lox^;Nestin^Cre^* neocortex, while the number of symmetric proliferative divisions were three-fold reduced (p<0.05, Fig. 2G). Moreover, the numbers of both symmetric proliferative and neurogenic divisions in the *Ptc1^Lox/Lox^;Rbpj^Lox/Lox^;Nestin^Cre^* neocortex were comparable to the wild type. Overall, these results suggest that although the disorganisation of the neocortical structure was not completely rescued (Fig. 4), inactivation of the Notch signaling pathway in Hh-activated progenitors restores the balance between symmetric neurogenic and proliferative divisions in the mutants to wild type levels.

In addition to the Hh pathway, the canonical Rbpj mediated Notch pathway has been shown to regulate neural stem cells [17]. To investigate the effect of loss of Notch signaling on Hh-activated neocortical progenitor cells, we utilised the SFUA. As described previously, an 8-fold increase in sphere forming potential was observed between wild type and *Ptc1^Lox/Lox^;Nestin^Cre^* neocortex (Fig. 2A). Loss of both *Rbpj* and *Ptc1* alleles in Nestin^+^ progenitors led to a 4.3-fold reduction (p = 0.0200) in the sphere forming capacity of neocortical cells (0.500±0.094%) compared to *Ptc1^Lox/Lox^;Nestin^Cre^* neocortex (2.154±0.267%). Moreover, these levels were comparable to the sphere forming potential of wild type neocortex. Interestingly, a 4.6-fold increase in sphere forming potential was observed in *Ptc1^Lox/Lox^;Rbpj^Lox/Lox^;Nestin^Cre^* neocortex compared to *Rbpj^Lox/Lox^;Nestin^Cre^* neocortex (p = 0.0427) suggesting that Hh signaling can compensate for loss of Notch signaling to support the sphere forming properties of neocortical progenitors (Fig. 2A). To further examine whether loss of *Rbpj* regulates *Ptc1* deleted neocortical stem cells, the neurosphere assay was performed on neocortical samples. As detailed previously, a significant increase in the rate of clonal expansion of *Ptc1^Lox/Lox^;Nestin^Cre^* neocortex stem cells was observed compared to the *Ptc1^Lox/Lox^* neocortex (0.8877±0.06774 vs. 0.7060±0.03302, p<0.05; Fig. 2B). In contrast, stem cells from *Rbpj^Lox/Lox^;Nestin^Cre^* neocortex failed to expand clonally in the neurosphere assay post passage 1 (n = 3), although live cells were observed in the culture up to passage 4 (Fig. 2B, Fig. S7B). The rate of clonal expansion of *Rbpj^Lox/Lox^;Nestin^Cre^* neocortex was significantly reduced (−1.337±3.400e+038, p<0.0001) compared to *Ptc1^Lox/Lox^* wild type neocortex (Fig. 2B). Homozygous loss of *Rbpj* in addition to loss of *Ptc1* led to a significant reduction in the number of stem cells (−0.08099±0.04816) compared to wild type samples. Moreover, an apparent reduction in neurosphere size and altered morphology in comparison to neurosphere cultures derived from the wild type neocortex was noted (Fig. S7B), possibly due to failure of stem cells to survive. Taken together, these data suggest that homozygous loss of *Rbpj*, irrespective of the presence or absence of *Ptc1*, ultimately results in loss of clonal expansion of neural stem cells *in vitro*.

### Some Notch effectors are upregulated in Hedgehog activated ventricular zone cells

**Figure 5:**
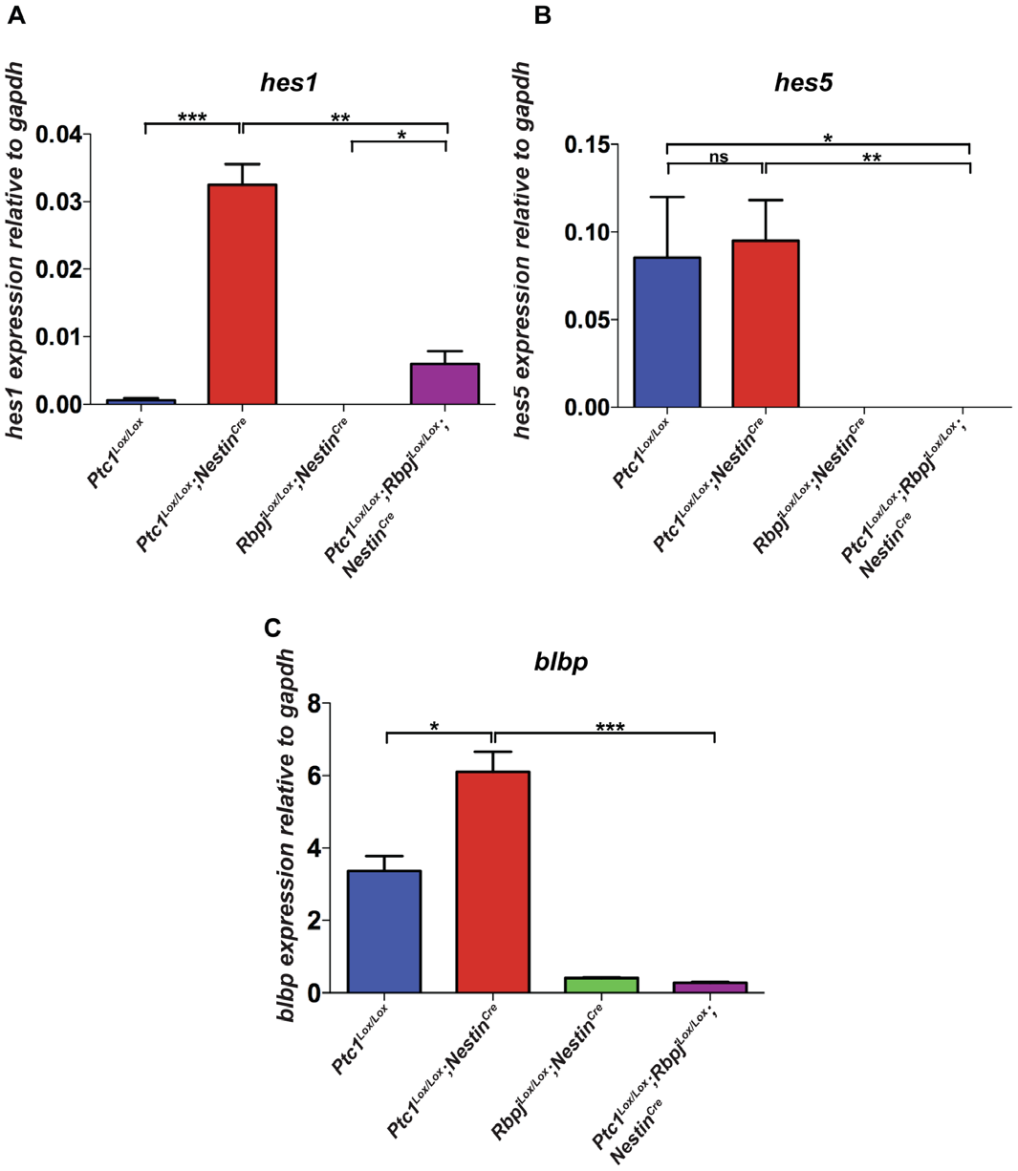
Inactivation of Ptc1 results in regulation of downstream targets of Notch signaling at E14.5. (**A,** **B, C**): Taqman RT-PCR for detection of *Hes1*, *Hes5* and *Blbp* transcript from microdissected VZ (n≥3 for each genotype). *Hes1* transcript was 65-fold upregulated in *Ptc1^Lox/Lox^;Nestin^Cre^* neocortex compared to *Ptc1^Lox/Lox^* VZ (**A**) and *Blbp* transcript was approximately two-fold upregulated compared to wild type VZ (p<0.05) (**C**). The expression of *Hes1*, *Blbp* and *Hes5* was significantly downregulated following deletion of Rbpj compared to *Ptc1* deletion alone (p<0.05). The data were normalised based on the expression of *Gapdh.*

Clearly, our data indicate that Hedgehog pathway and Notch pathway activity can co-operate to regulate neurogenic divisions of neocortical progenitors. Therefore, we next examined whether the expansion of progenitor cells and the concomitant depletion of neurons in the VZ of E14.5 *Ptc1^Lox/Lox^;Nestin^Cre^* mice was potentially the result of the upregulation of Notch signaling. Figure 5A demonstrates that in wild type VZ *Hes1* expression is low, and undetectable in the absence of Rbpj as expected. In the presence of Hedgehog pathway activation *Hes1* activity is high but this activity is largely lost in the absence of Rbpj, suggesting that most of the Hedgehog-dependent *Hes1* activity is dependent upon active canonical signaling. Interestingly, there is a minor component of Hh-dependent *Hes1* activity that is not dependent upon Notch signaling, consistent with our results from the VZ of the cerebellum where there is also a Hedgehog and Notch-dependent *Hes1* activity, and the data of others [46], [47], [48]. Overall, the *Hes1* data indicate that in the developing VZ of the neocortex, Hedgehog pathway activation positively regulates *Hes1* via Rbpj. Similarly, we analyzed *Blbp* expression (Figure 5C). Blbp has been shown to be a canonical Notch target in radial glial cells in the VZ [18]. Similar to *Hes1*, *Blbp* shows a distinct upregulation in response to activation of the Hedgehog pathway, and this activation is abrogated by loss of Rbpj. A third potential Notch effector in the VZ, *Hes5* was also examined (Fig. 5B). From these data it can be observed that *Hes5* expression requires Rbpj, as expected, but does not respond to Hedgehog pathway activation. On the whole, the data presented in Figure 5 suggest that the undoubted interaction observed at the developmental and stem cell level between Hedgehog and Notch signaling is reflected in part by an apparent Rbpj-dependent regulation by Hedgehog signaling of some, but not all, Notch effectors in the VZ.

## Discussion

The processes that direct the neuroepithelium to generate cerebral cortex are clearly central to the functioning of higher mammals. The Hedgehog pathway has long been recognized to play a key role in the development of ventral aspects of the CNS and the maintenance of neural stem cells more broadly. Recently, Shh loss of function analyses have indicated that Hh signaling is required for the correct establishment of dorsal patterning in the forebrain, with the window of activity likely to extend at the least from E12.5–E15.5, a critical period for corticogenesis [12]. Similarly, regulation of Notch signaling is essential for neurogenesis with activated Notch signaling being required for the maintenance of a progenitor fate in aspects of the CNS, including the VZ of the forebrain [13], [14], [19]. Here, we used a genetic approach to examine the interaction between Hedgehog and Notch signaling in the neocortex. Activity of the Hh pathway is dependent upon the interaction between Ptc1 and Smo, with the activity of Hedgehog ligands serving to disrupt that relationship. At the molecular level, Hh signaling is generated from unrestrained Smo, not the Hedgehog ligand *per se*. Ptc1 is therefore a negative regulator of Hh signaling and here we have demonstrated that loss of Ptc1 in Nestin+ neuroepithelial cells and progeny leads to sustained, cell autonomous signaling from E11.5/E12.5 (Fig. 1 and data not shown). By taking this approach we were able to model a high level of Hh signal activation over a time window reflecting the expression and activity of endogenous Shh ligand, which continues at least until E15.5 [12]. It is clear from our data that this model likely generates a level of Hedgehog signal and response in excess to that of the endogenous Shh ligand, which is present in the VZ, SVZ and cortical plate. This approach enabled us to effectively address two issues. First, although loss of function studies have suggested that Hh signaling is a required mitogen for stem cells of the neocortex (and tectum), and *in vitro* activation studies have suggested the same [10], [11], [12]. Our model enabled us for the first time to examine the consequences of Hh activation *in vivo*, particularly in the control of symmetric/asymmetric stem cell divisions of VZ progenitor cells. Secondly, loss of Hedgehog function in the developing dorsal CNS creates severe phenotypes which have made interpretation difficult when superimposing other genotypes, and the gain of Hh function phenotype we define here has enabled the consequences of concomitant loss of Notch function to be readily assessed.

Notch signaling utilizes a membrane bound ligand that contacts an appropriate receptor leading to the release of a cytoplasmic fragment of the receptor which then forms a multi-unit complex capable of transcriptional regulation. In mammals, ligands include members of the Delta-like (DLL1, DLL3, DLL4) and Jagged (Jag1, Jag2) families, and receptors include Notch1-4. Of the receptors, at least Notch1-3 are expressed and functional in the developing forebrain and of the ligands, Jag1 and DLL1, 3 [49], [50], [51]. Although the potential combinatorial complexity of Notch ligand/receptor interaction in the developing forebrain is high, canonical Notch signaling requires the CSL/Rbpj (Rbpj) transcription co-factor. So in practice, inhibition of Rbpj function will abrogate Notch receptor signaling [52]. Accordingly, here we took the approach of attenuating Notch signaling by deleting Rbpj, which has proved to be a highly effective approach in the developing forebrain and other tissues [17], [25], [52]. In the absence of the Notch ICD the interaction of Rbpj with target promoters is thought to be transient and weak, nonetheless it is possible that deletion of Rbpj may itself lead to “de-repression” of some Notch target genes but the neocortical phenotype we observed in this study is consistent with data indicating a loss of Notch activity [39], [40], [53].

Following the loss of Ptc1 under the control of *Nestin^Cre^*, we observed a robust Hh response from E11.5 in the neocortex as indicated by the upregulation of *Gli1* and *Ptc1* itself. At the gross anatomical level this resulted in an irregular and folded neocortex by E14.5 and more detailed analysis indicated an expanded VZ/SVZ region with a high mitotic index, with the neurosphere assay indicating this region contained an increased number of stem/progenitor cells. It has been known for some time that neocortical stem/progenitor cells can be expanded *in vitro* by the addition of Shh [20], [21] and here we have demonstrated that ligand-independent activation of the Hh pathway can produce a similar expansion *in vivo*. Clonal expansion studies of neurosphere cultures are one measure of the long-term proliferative potential of stem cells and our data indicate that the Hh pathway is mitogenic for such cells. The progenitor cell in the neocortex is the radial glial (RG) cell, which also acts physically as a migratory scaffold for differentiating cells to populate the dorsal layers with the majority of the neurons in the brain being derived directly or indirectly (through basal progenitors) from radial glial cells [2], [54], [55], [56], [57], [58]. Studies described here have demonstrated that TuJ1^+^ neurons were also positive for GLAST suggesting that majority of neurons originated from the radial glial progenitor cell. We quantified the consequences of Hh pathway activation in Nestin^+^ progenitors on the fate of RG cells using the pair cell assay and determined that at E14.5 there was an increase in the number of symmetric proliferative divisions at the expense of both neurogenic and differentiative divisions, with immunohistochemical data supporting those conclusions. Even though previous studies have suggested that Hh is a mitogen *in vitro* for neural stem cells, our data indicate that Hh activation can not only increase the number of stem/progenitor cells *in vivo*, but in doing so shifts the balance of RG cell neurogenic divisions. The mechanisms that underlie the switch from symmetric proliferative to symmetric/asymmetric neurogenic divisions are poorly understood. According to the cell cycle length hypothesis, lengthening of the cell cycle allows a neurogenic cell fate determinant sufficient time to induce a cell fate change resulting in differentiation [59]. Consistent with this hypothesis, here we demonstrate that *Ptc1*-inactivated neocortical VZ progenitors cycle more rapidly, and fail to exit the cell cycle, potentially accounting for the decrease in neurogenic divisions. During development it is likely the basal low level of Ptc1 expression in radial glial cells is sufficient to antagonise Hh pathway activity to preserve the balance between stem cell maintenance and neurogenesis.

Several reports have suggested the importance of Notch signaling in gliogenesis [13], [60] and *in vivo* and *in vitro* studies have established that Notch 1 and Notch 3 activation promotes radial glial cell fate [14], [39], similar to our findings with Hh pathway activation. Indeed, in a number of tissues such as cerebellum and skin [26], [61] there appears to be interaction between the Hh and Notch pathways. For example, in the cerebellum it has been demonstrated that Notch1 controls cerebellar granule-cell proliferation and differentiation [62], as does Sonic Hedgehog [63]. Furthermore, the elevation of Notch2 and Hes5 in Smoothened-induced medulloblastomas suggests that Hh pathway activation interacts with Notch signaling [64]. Apart from the brain, deregulation of Hh, Notch and Bmi pathways has been proposed to induce transformation of mammary stem cells [65]. Taken together, these reports provide evidence in favour of interplay between the Hh and Notch signaling pathways. The results presented here demonstrate that activation of the Hh signaling pathway by *Ptc1* deletion increases the stemness of radial glial cells likely by upregulating the Notch signaling pathway. Further, concomitant inactivation of *Rbpj* in Hh-activated neural progenitors restored the balance between symmetric proliferative and neurogenic divisions, thereby suggesting a crosstalk between the Hh and Notch signaling pathways during normal corticogenesis.

The transcription factor Hes1 is possibly a key molecule in the Hh/Notch co-regulation of corticogenesis. Hes1 is generally regarded as a canonical Notch pathway effector and it is essential for maintaining neural stem cells through out the CNS, including the developing dorsal structures [66]. However, early in neuroepithelial development there appears to be instances where *Hes1* expression is likely not dependent upon Notch signaling [67]. Furthermore, studies have shown that *Hes1* may be regulated by Shh without the requirement for Notch signaling in cerebellar granule neurons and mesodermal cells [46], [47]. In the retina, progenitor cell proliferation is under the control of Notch-independent Sonic hedgehog/Hes1 activity and the Hh signal effector Gli2 binds directly to the *Hes1* promoter [48]. Here, our data in the neocortex similarly suggests that *Hes1* is upregulated by Hh signaling but is not influenced by concomitant loss of Rbpj, consistent with our findings in the ventricular zone of the cerebellum [61]. We would therefore suggest that *Hes1* is not only a critical regulator of neuronal cell fate in multiple niches but also uniquely represents a molecular convergence point between Hh and Notch signaling in those niches, and an important avenue for further study.

Although there have been many studies conducted on the role of Notch signaling in radial glial cells, the crosstalk between the Notch and Hh signaling pathways in controlling progenitor cell expansion is still poorly understood. As radial glial cells play a central role in the cytoarchitecture of the neocortex acting both as migratory scaffolds and glial progenitors, the understanding of radial glial development is crucial for treatment of both developmental and degenerative disorders. Specifically, *in vivo* approaches have demonstrated that administration of Notch ligand to stimulate the neural stem cell niche has been of direct therapeutic benefit in animal models of ischaemic brain injury [68] and Parkinson's disease [69]. On the basis of our data we suggest that agonists of the Hedgehog pathway alone or in combination with Notch pathway manipulation may also be a novel and useful therapeutic approach to stimulating neuronal cell niches *in vivo* as well as *in vitro*.

## Supporting Information

Figure S1 Genotyping of microdissected VZ and Real Time PCR analysis. Genotyping of microdissected E14.5 VZ demonstrates that *Ptc1* is inactivated (as indicated by the loss of the wild type 450 bp transcript) upon Nestin Cre-mediated recombination (A). (B, C): SYBR Green RT-PCR for detection of *Ptc1* deleted transcript and Taqman RT-PCR for detection of *Gli1* transcript. *Ptc1* transcript was downregulated and *Gli1* upregulated (C) in the *Ptc1^Lox/Lox^;Nestin^Cre^* VZ compared wildtype E14.5 *Ptc1^Lox/Lox^* VZ, as a result of Cre-mediated recombination. The data for *Ptc1* and *Gli1* was normalised based on the expression of *Hprt* and *Gapdh* respectively, and is presented as two independent pooled samples of each genotype.
(0.94 MB TIF)

Figure S2 The irregularities in the thickness within the *Ptc1^Lox/Lox^;Nestin^Cre^* neocortex do not change in severity depending on the rostral/caudal positioning. E14.5 Haematoxylin and Eosin stained coronal sections at rostral (A, B), intermediate rostral-caudal (C, D), and caudal levels (E, F) of the *Ptc1^Lox/Lox^;Nestin^Cre^* neocortex. Scale bar (A–F), 500 µm.
(2.85 MB TIF)

Figure S3 Shortening of cell cycle and increased re-entry in *Ptc1^Lox/Lox^;Nestin^Cre^* neocortex. Co-immunofluorescence analysis of BrdU and Ki67 expression in coronal sections of E14.5 neocortices. In the mutant neocortex, there is an increase in progenitor cells (Ki67^+^) undergoing S-phase (BrdU^+^) compared to the wildtype neocortex (A). Quantification of the percentage of progenitor cells (Ki67^+^, red) co-labelled with BrdU (green) after a 2 hour pulse label (B). Rate of cell division is increased in E14.5 *Ptc1^Lox/Lox^;Nestin^Cre^* neocortex (n = 3; 83±1.4; p<0.0001) as compared to *Ptc1^Lox/Lox^* wild type littermates (n = 3; 61±2.0) indicating mutant cells cycle faster. Cells re-entering the cell cycle are BrdU^+^/Ki67^+^ whereas cells no longer dividing and withdrawn from cell cycle are BrdU^+^/Ki67^−^ after a single pulse label of BrdU 24 hours prior to being killed (C). Approximately twice as many wild type progenitors leave the cell cycle, as compared to *Ptc1^Lox/Lox^;Nestin^Cre^* progenitors (n = 3; p<0.0001) (D). DNA is stained with DAPI. Scale bar (A), 200 µm; (C), 50 µm. Co-immunofluorescence of GLAST (red) and Tbr2 (green) (E, F) on neocortical progenitors isolated from E14.5 *Ptc1^Lox/Lox^;Nestin^Cre^* and *Ptc1^Lox/Lox^* neocortex. GLAST^+^ radial glial cells from *Ptc1^Lox/Lox^;Nestin^Cre^* neocortex rarely differentiate into Tbr2^+^ basal progenitors after 24 hours (F, G). Arrowhead (in E) indicates Tbr2^+^ cells. Quantitative analysis for GLAST and Tbr2 (G) revealed that the percentage of RG cells undergoing symmetric differentiative divisions (1RG = 2 Basal progenitor (BP) cells) was significantly reduced in the *Ptc1^Lox/Lox^;Nestin^Cre^* neocortex. In addition, reduction in the percentage of RG cells undergoing asymmetric differentiative (1RG = RG+BP) divisions was significant (G). Bars represent standard errors. **p<0.05. Scale bar (E, F), 10 µm.
(4.99 MB TIF)

Figure S4 The radial glial progenitor population is expanded in *Ptc1^Lox/Lox^;Nestin^Cre^* neocortex (B). Nestin expression, which is a ubiquitous marker for neural progenitors, is expanded at E14.5 in the *Ptc1^Lox/Lox^;Nestin^Cre^* VZ (B) as compared to *Ptc1^Lox/Lox^* neocortex (A). Assessment of neuronal differentiation in the wild type and mutant neocortex. At E12.5 a layer of MAP2^+^ neurons emerges beneath the pial surface in both the *Ptc1^Lox/Lox^* neocortex (C) and *Ptc1^Lox/Lox^;Nestin^Cre^* neocortex (D). BrdU-birthdating of neurons was performed by injecting pregnant females with BrdU at E11.5 (E, F) or E12.5 (G, H) and sacrificing at E14.5. BrdU immunostaining revealed that radial distribution of neurons born at E11.5 (E, F) or E12.5 (G, H) is indistinguishable at E14.5 between the *Ptc1^Lox/Lox^* neocortex (E, G) and *Ptc1^Lox/Lox^;Nestin^Cre^* neocortex (F, H). Abbreviations: CP, cortical plate; IZ, intermediate zone; MZ, marginal zone; PP, pre-plate; SP, sub-plate; SVZ, sub-ventricular zone; VZ, ventricular zone. Scale bar (A–B, E–H), 20 µm; (C, D), 10 µm.
(6.05 MB TIF)

Figure S5 Disruption of neocortical structure in the *Rbpj^Lox/Lox^;Nestin^Cre^* mice. (A, B): Haematoxylin and Eosin stained coronal sections of E14.5 neocortex. (B): shows a higher magnification of the mutant neocortex in (A). The neocortex is highly disorganised, lacks diencephalon and contains blood clots (arrowheads) within the neocortex and the LV. (C): Nissl-stained coronal sections of E14.5 neocortices. Scale bar, 100 µm. Abbreviations: LV, lateral ventricle; NC, neocortex.
(6.60 MB TIF)

Figure S6 Loss of Rbpj leads to disruption of the neocortical patterning. Immunostainingof E14.5 neocortex using TuJ1 (red) (A–D); (green) (E–H) and Nestin (green) (A–D) or Sox2 (red) (E–H) antibodies and counterstained with DAPI (blue). Confocal images of medial region of the coronal sections are shown. Abbreviations: CP, cortical plate; VZ, ventricular zone. Scale bar (A–H), 20 µm.
(9.19 MB TIF)

Figure S7 Neurosphere assay of mutant neocortices compared to wild type spheres. (A–D): Bright field microscope images of passage 7 neurosphere cultures (A, C, D) and passage 1 (B). Inset images for (A–D) reveal microspikes on *Ptc1^Lox/Lox^* neurospheres while homozygous loss of Rbpj results in no sphere generation (C, D). Scale bar (A–D), 10 µm.
(2.60 MB TIF)
