# Supplementary figures and images for: Sonic Hedgehog and Notch Signaling Can Cooperate to Regulate Neurogenic Divisions of Neocortical Progenitors
Source: PLoS One. 2011 Feb 17;6(2):e14680. doi: 10.1371/journal.pone.0014680 (PMC3040755; doi:10.1371/journal.pone.0014680)

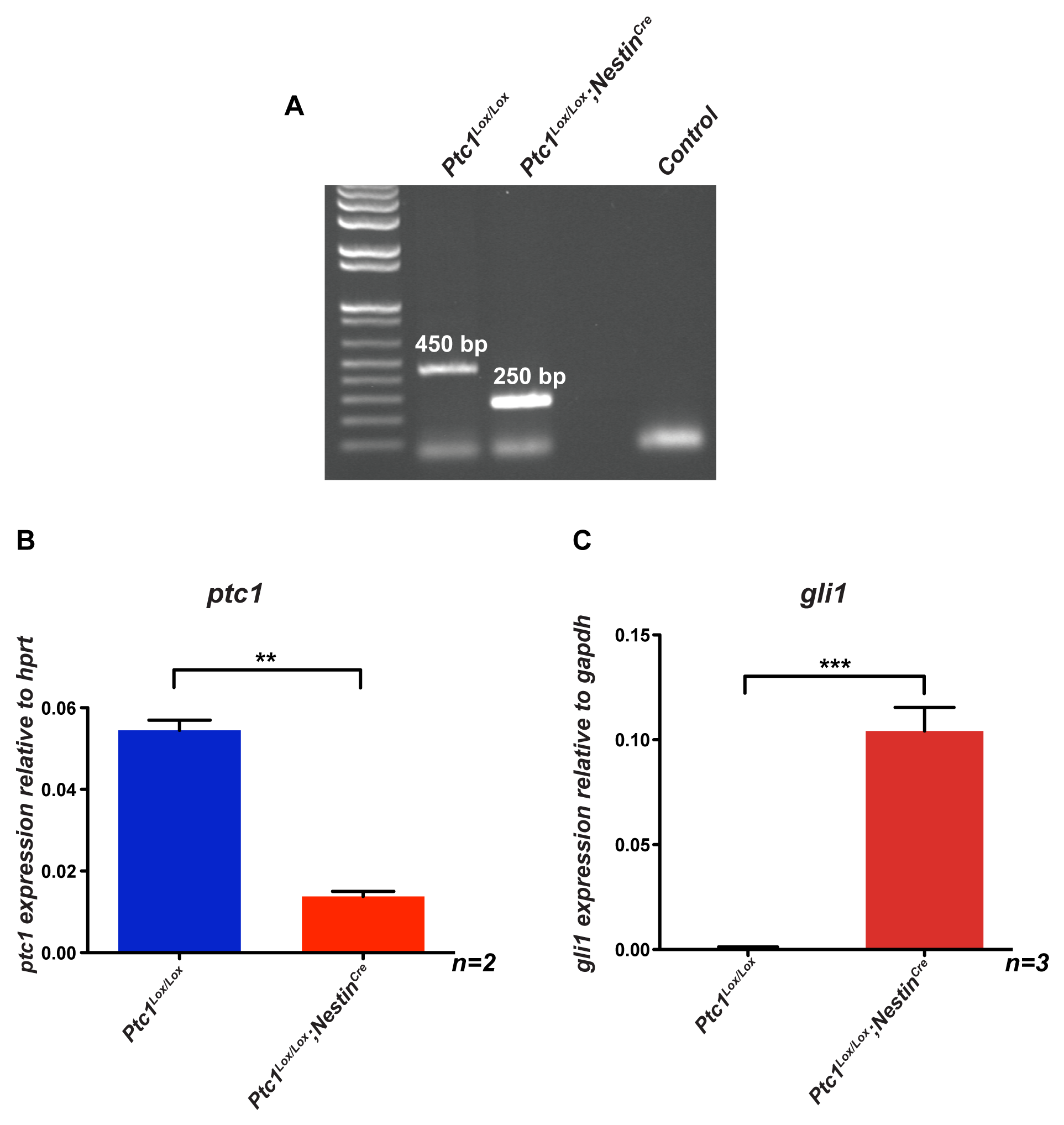

Supplement: Figure S1 — Genotyping of microdissected VZ and Real Time PCR analysis. Genotyping of microdissected E14.5 VZ demonstrates that Ptc1 is inactivated (as indicated by the loss of the wild type 450 bp transcript) upon Nestin Cre-mediated recombination (A). (B, C): SYBR Green RT-PCR for detection of Ptc1 deleted transcript and Taqman RT-PCR for detection of Gli1 transcript. Ptc1 transcript was downregulated and Gli1 upregulated (C) in the Ptc1Lox/Lox;NestinCre VZ compared wildtype E14.5 Ptc1Lox/Lox VZ, as a result of Cre-mediated recombination. The data for Ptc1 and Gli1 was normalised based on the expression of Hprt and Gapdh respectively, and is presented as two independent pooled samples of each genotype. (0.94 MB TIF) [file pone.0014680.s001.tif]

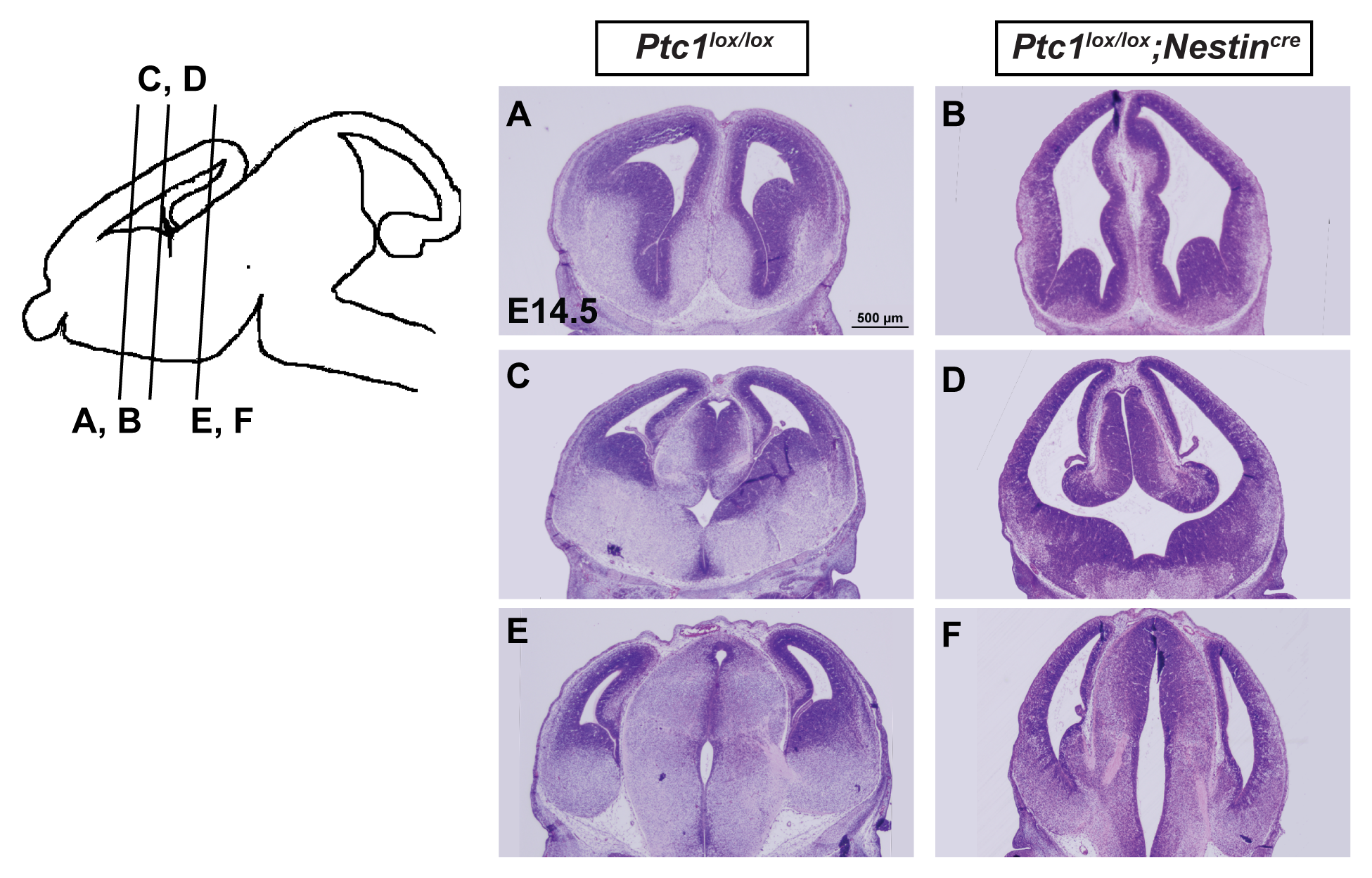

Supplement: Figure S2 — The irregularities in the thickness within the Ptc1Lox/Lox;NestinCre neocortex do not change in severity depending on the rostral/caudal positioning. E14.5 Haematoxylin and Eosin stained coronal sections at rostral (A, B), intermediate rostral-caudal (C, D), and caudal levels (E, F) of the Ptc1Lox/Lox;NestinCre neocortex. Scale bar (A–F), 500 µm. (2.85 MB TIF) [file pone.0014680.s002.tif]

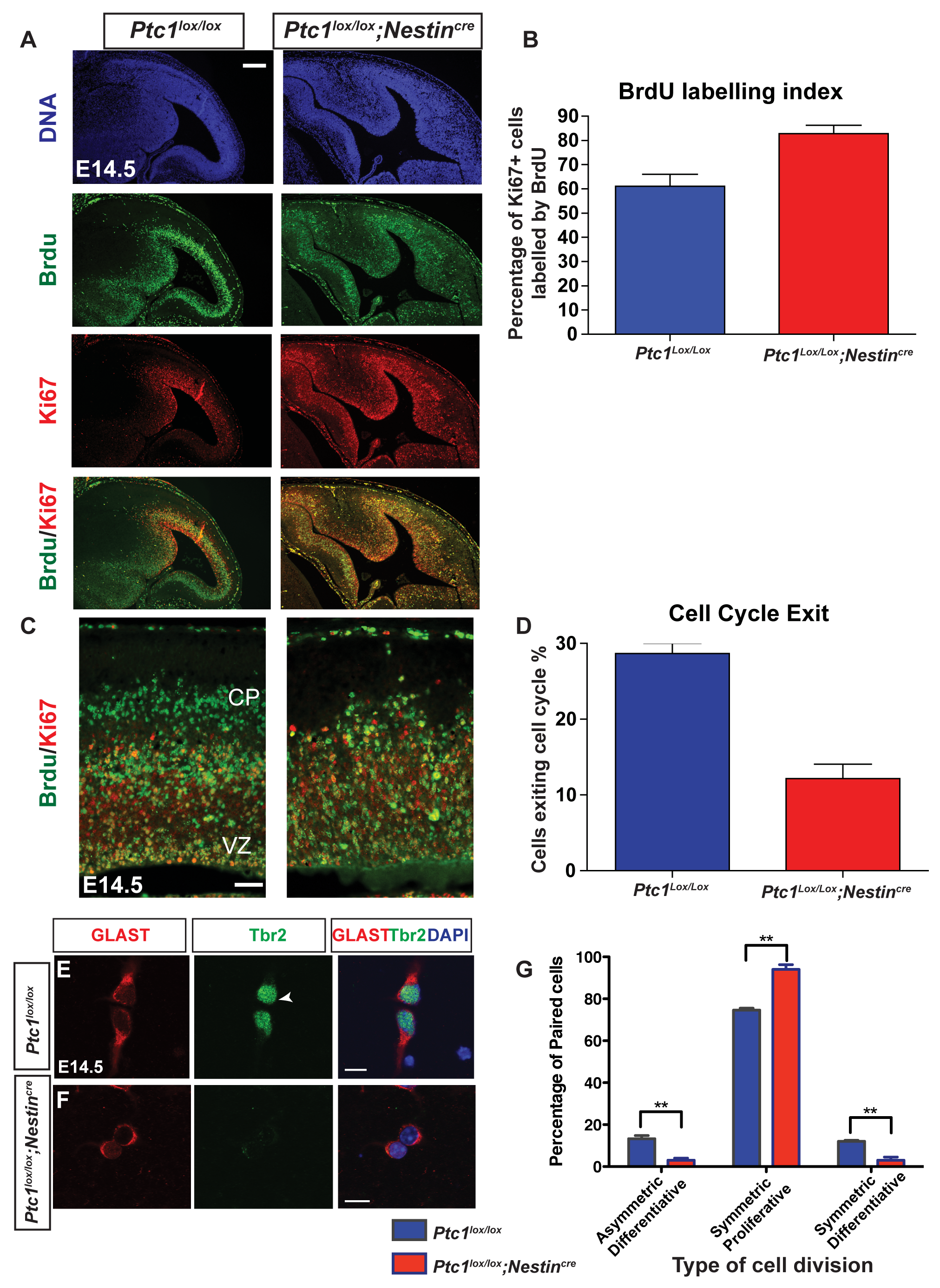

Supplement: Figure S3 — Shortening of cell cycle and increased re-entry in Ptc1Lox/Lox;NestinCre neocortex. Co-immunofluorescence analysis of BrdU and Ki67 expression in coronal sections of E14.5 neocortices. In the mutant neocortex, there is an increase in progenitor cells (Ki67+) undergoing S-phase (BrdU+) compared to the wildtype neocortex (A). Quantification of the percentage of progenitor cells (Ki67+, red) co-labelled with BrdU (green) after a 2 hour pulse label (B). Rate of cell division is increased in E14.5 Ptc1Lox/Lox;NestinCre neocortex (n = 3; 83±1.4; p<0.0001) as compared to Ptc1Lox/Lox wild type littermates (n = 3; 61±2.0) indicating mutant cells cycle faster. Cells re-entering the cell cycle are BrdU+/Ki67+ whereas cells no longer dividing and withdrawn from cell cycle are BrdU+/Ki67− after a single pulse label of BrdU 24 hours prior to being killed (C). Approximately twice as many wild type progenitors leave the cell cycle, as compared to Ptc1Lox/Lox;NestinCre progenitors (n = 3; p<0.0001) (D). DNA is stained with DAPI. Scale bar (A), 200 µm; (C), 50 µm. Co-immunofluorescence of GLAST (red) and Tbr2 (green) (E, F) on neocortical progenitors isolated from E14.5 Ptc1Lox/Lox;NestinCre and Ptc1Lox/Lox neocortex. GLAST+ radial glial cells from Ptc1Lox/Lox;NestinCre neocortex rarely differentiate into Tbr2+ basal progenitors after 24 hours (F, G). Arrowhead (in E) indicates Tbr2+ cells. Quantitative analysis for GLAST and Tbr2 (G) revealed that the percentage of RG cells undergoing symmetric differentiative divisions (1RG = 2 Basal progenitor (BP) cells) was significantly reduced in the Ptc1Lox/Lox;NestinCre neocortex. In addition, reduction in the percentage of RG cells undergoing asymmetric differentiative (1RG = RG+BP) divisions was significant (G). Bars represent standard errors. **p<0.05. Scale bar (E, F), 10 µm. (4.99 MB TIF) [file pone.0014680.s003.tif]

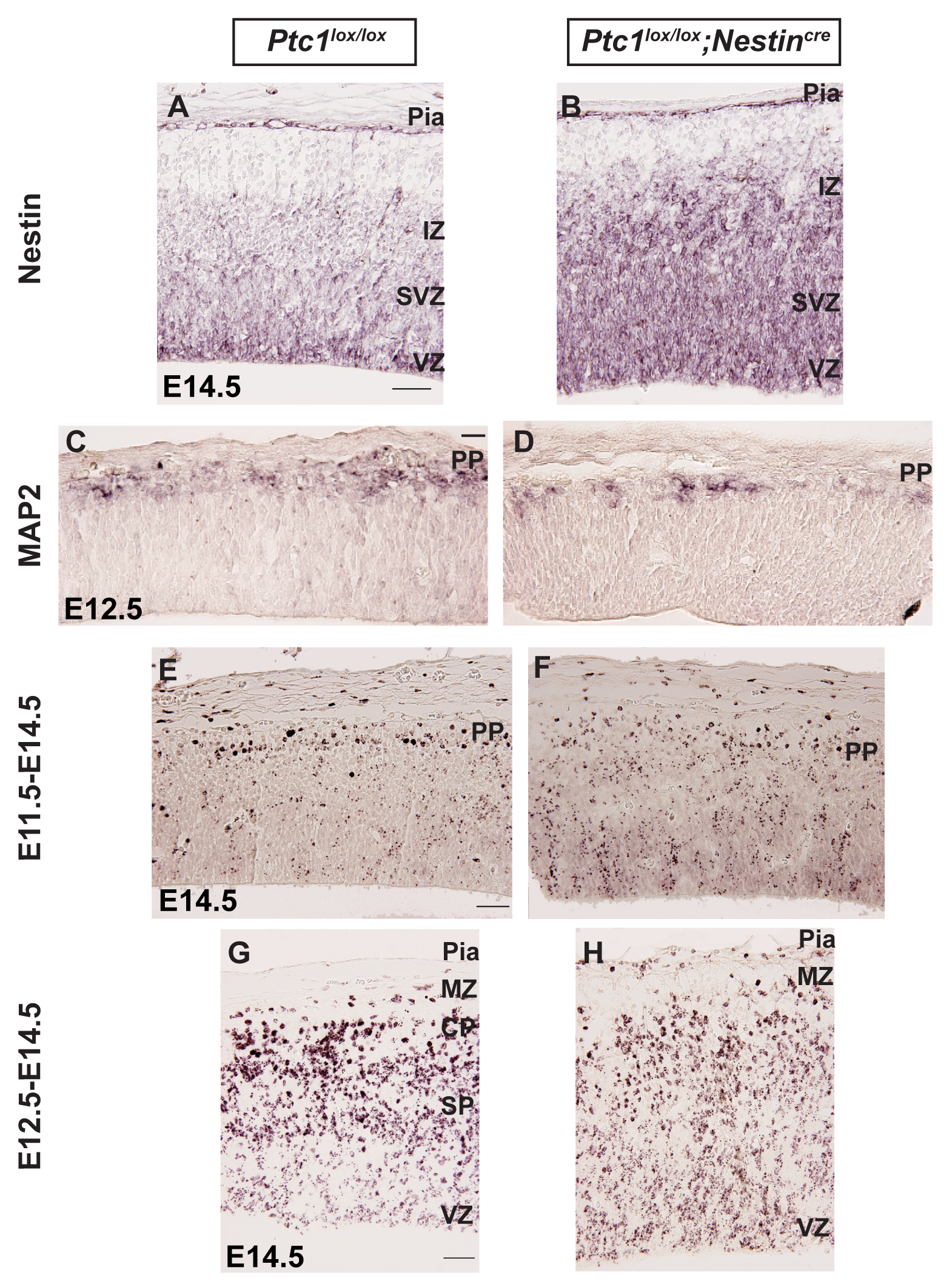

Supplement: Figure S4 — The radial glial progenitor population is expanded in Ptc1Lox/Lox;NestinCre neocortex (B). Nestin expression, which is a ubiquitous marker for neural progenitors, is expanded at E14.5 in the Ptc1Lox/Lox;NestinCre VZ (B) as compared to Ptc1Lox/Lox neocortex (A). Assessment of neuronal differentiation in the wild type and mutant neocortex. At E12.5 a layer of MAP2+ neurons emerges beneath the pial surface in both the Ptc1Lox/Lox neocortex (C) and Ptc1Lox/Lox;NestinCre neocortex (D). BrdU-birthdating of neurons was performed by injecting pregnant females with BrdU at E11.5 (E, F) or E12.5 (G, H) and sacrificing at E14.5. BrdU immunostaining revealed that radial distribution of neurons born at E11.5 (E, F) or E12.5 (G, H) is indistinguishable at E14.5 between the Ptc1Lox/Lox neocortex (E, G) and Ptc1Lox/Lox;NestinCre neocortex (F, H). Abbreviations: CP, cortical plate; IZ, intermediate zone; MZ, marginal zone; PP, pre-plate; SP, sub-plate; SVZ, sub-ventricular zone; VZ, ventricular zone. Scale bar (A–B, E–H), 20 µm; (C, D), 10 µm. (6.05 MB TIF) [file pone.0014680.s004.tif]

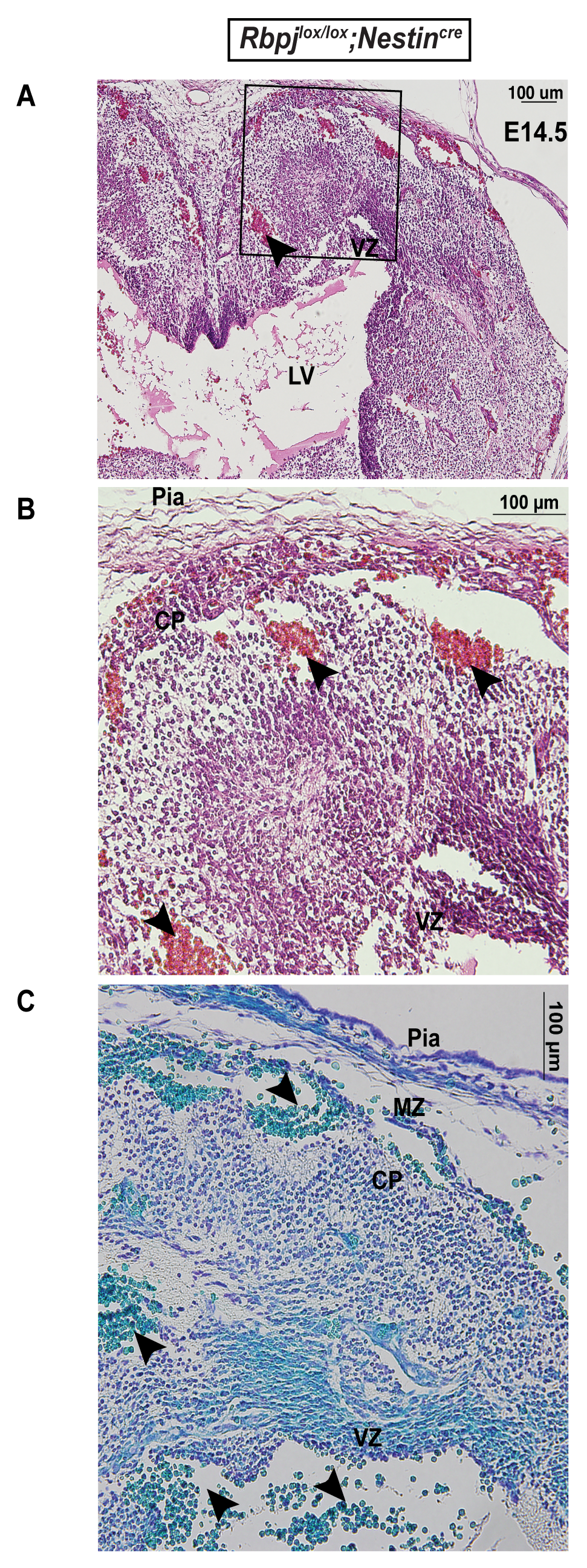

Supplement: Figure S5 — Disruption of neocortical structure in the RbpjLox/Lox;NestinCre mice. (A, B): Haematoxylin and Eosin stained coronal sections of E14.5 neocortex. (B): shows a higher magnification of the mutant neocortex in (A). The neocortex is highly disorganised, lacks diencephalon and contains blood clots (arrowheads) within the neocortex and the LV. (C): Nissl-stained coronal sections of E14.5 neocortices. Scale bar, 100 µm. Abbreviations: LV, lateral ventricle; NC, neocortex. (6.60 MB TIF) [file pone.0014680.s005.tif]

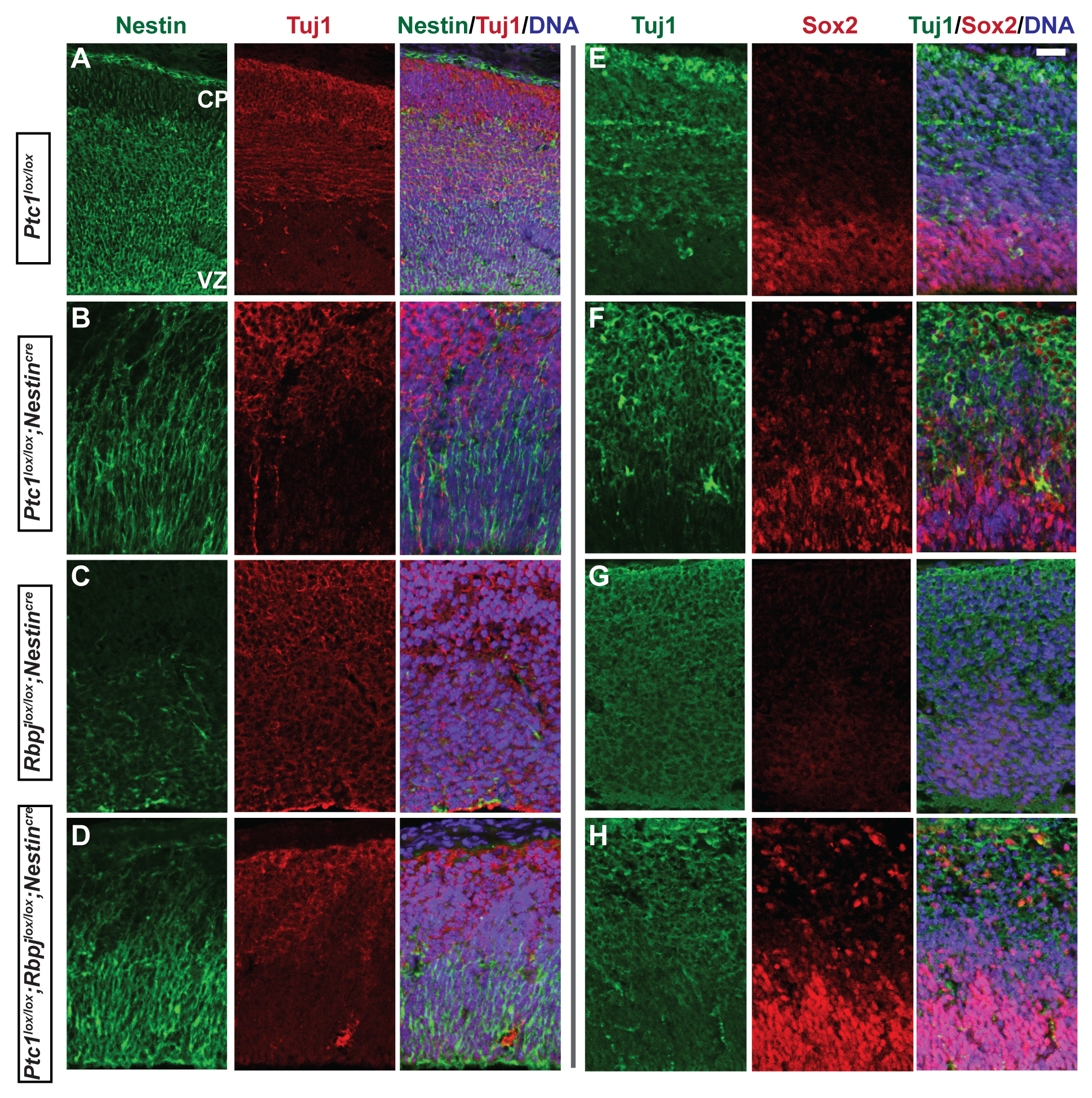

Supplement: Figure S6 — Loss of Rbpj leads to disruption of the neocortical patterning. Immunostainingof E14.5 neocortex using TuJ1 (red) (A–D); (green) (E–H) and Nestin (green) (A–D) or Sox2 (red) (E–H) antibodies and counterstained with DAPI (blue). Confocal images of medial region of the coronal sections are shown. Abbreviations: CP, cortical plate; VZ, ventricular zone. Scale bar (A–H), 20 µm. (9.19 MB TIF) [file pone.0014680.s006.tif]

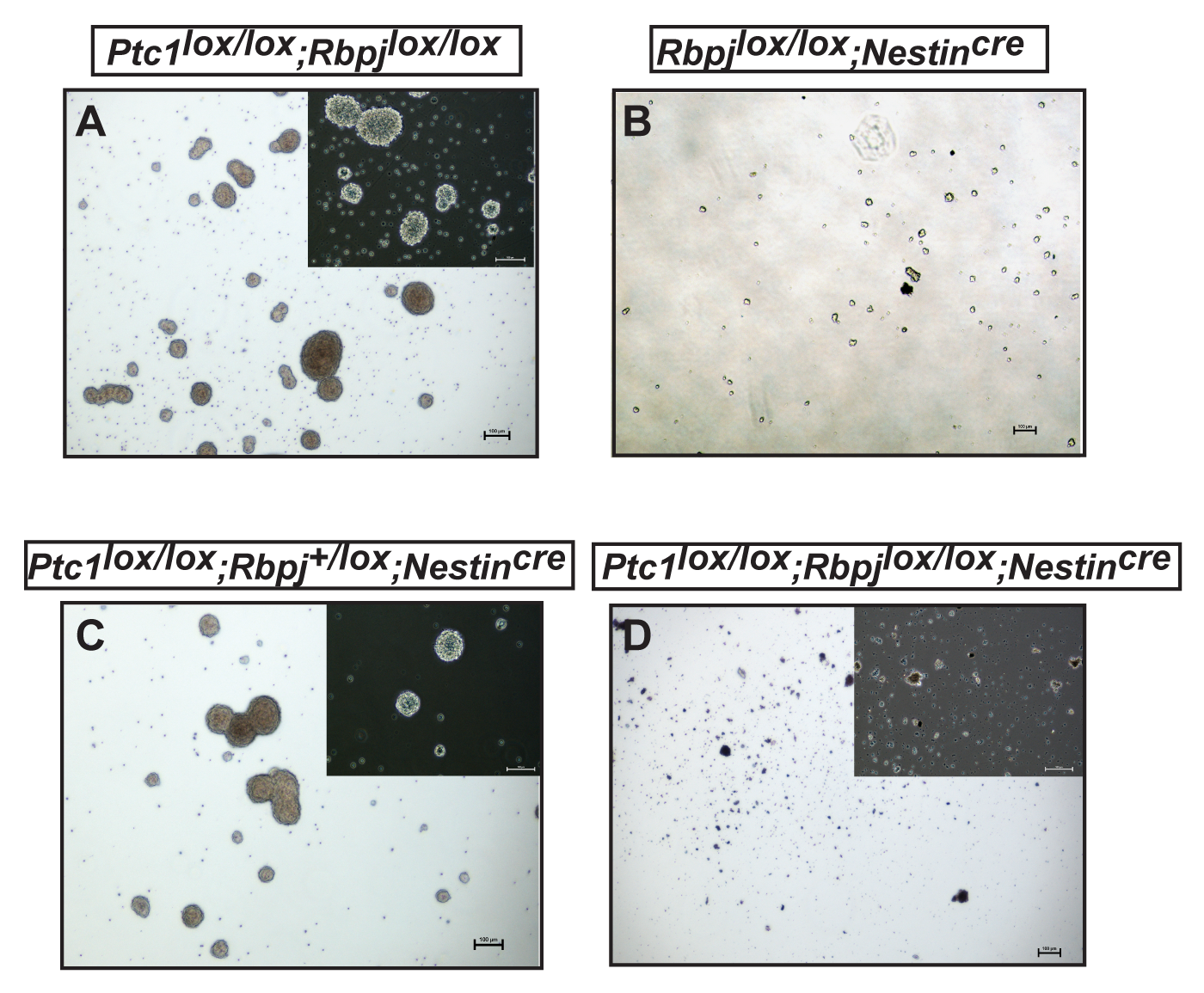

Supplement: Figure S7 — Neurosphere assay of mutant neocortices compared to wild type spheres. (A–D): Bright field microscope images of passage 7 neurosphere cultures (A, C, D) and passage 1 (B). Inset images for (A–D) reveal microspikes on Ptc1Lox/Lox neurospheres while homozygous loss of Rbpj results in no sphere generation (C, D). Scale bar (A–D), 10 µm. (2.60 MB TIF) [file pone.0014680.s007.tif]
